# Comparative analysis of whole plant, flower and root extracts of *Chamomilla recutita* L. and characteristic pure compounds reveals differential anti-inflammatory effects on human T cells

**DOI:** 10.3389/fimmu.2024.1388962

**Published:** 2024-04-24

**Authors:** Divya Lairikyengbam, Bernhard Wetterauer, Michael Schmiech, Beate Jahraus, Henning Kirchgessner, Pille Wetterauer, Karina Berschneider, Verena Beier, Beate Niesler, Emre Balta, Yvonne Samstag

**Affiliations:** ^1^ Section Molecular Immunology, Institute of Immunology, Heidelberg University Hospital, Heidelberg, Germany; ^2^ Institute of Pharmacy and Molecular Biotechnology, Heidelberg University, Heidelberg, Germany; ^3^ Institute of Experimental and Clinical Pharmacology, Toxicology and Pharmacology of Natural Products, University of Ulm, Ulm, Germany; ^4^ Department of Human Molecular Genetics, Heidelberg University Hospital, Heidelberg, Germany; ^5^ nCounter Core Facility, Institute of Human Genetics, Heidelberg University Hospital, Heidelberg, Germany

**Keywords:** *Chamomilla recutita* L. extracts, apigenin, chamazulene, α-bisabolol, primary human T cells, inflammation, immunomodulation, LC-MS/MS

## Abstract

**Introduction:**

Chronic inflammation is a hallmark of chronic wounds and inflammatory skin diseases. Due to a hyperactive and prolonged inflammation triggered by proinflammatory immune cells, transitioning to the repair and healing phase is halted. T cells may exacerbate the proinflammatory milieu by secreting proinflammatory cytokines. *Chamomilla recutita* L. (chamomile) has been suggested for use in several inflammatory diseases, implying a capability to modulate T cells. Here, we have characterized and compared the effects of differently prepared chamomile extracts and characteristic pure compounds on the T cell redox milieu as well as on the migration, activation, proliferation, and cytokine production of primary human T cells.

**Methods:**

Phytochemical analysis of the extracts was carried out by LC-MS/MS. Primary human T cells from peripheral blood (PBTs) were pretreated with aqueous or hydroethanolic chamomile extracts or pure compounds. Subsequently, the effects on intracellular ROS levels, SDF-1α induced T cell migration, T cell activation, proliferation, and cytokine production after TCR/CD3 and CD28 costimulation were determined. Gene expression profiling was performed using nCounter analysis, followed by ingenuity pathway analysis, and validation at protein levels.

**Results:**

The tested chamomile extracts and pure compounds differentially affected intracellular ROS levels, migration, and activation of T cells. Three out of five differently prepared extracts and two out of three pure compounds diminished T cell proliferation. In line with these findings, LC-MS/MS analysis revealed high heterogeneity of phytochemicals among the different extracts. nCounter based gene expression profiling identified several genes related to T cell functions associated with activation and differentiation to be downregulated. Most prominently, apigenin significantly reduced granzyme B induction and cytotoxic T cell activity.

**Conclusion:**

Our results demonstrate an anti-inflammatory effect of chamomile- derived products on primary human T cells. These findings provide molecular explanations for the observed anti-inflammatory action of chamomile and imply a broader use of chamomile extracts in T cell driven chronic inflammatory diseases such as chronic wounds and inflammatory skin diseases. Importantly, the mode of extract preparation needs to be considered as the resulting different phytochemicals can result in differential effects on T cells.

## Introduction

Inflammation is a critical phenomenon during an immune response to an infection. However, when inflammation fails to resolve after clearing the infection, it becomes chronic, causing destruction of normal cells and tissues. Such a chronic inflammation is commonly observed in non-healing wounds in the skin, inflammatory skin diseases like psoriasis and atopic dermatitis and inflammatory bowel diseases like ulcerative colitis and Crohn’s disease. The prevalence of chronic inflammation is increasing due to different factors like lifestyle, food habits, aging, ultimately creating a huge socio-economic burden, and its effective treatments remains a challenge thus far (1, 2). In a normal wound healing process, there is a well-organized interplay between immune cells and non-immune cells, spread across four sequential, overlapping phases namely, homeostasis, inflammatory, proliferative and remodeling phases. An exacerbated inflammatory phase, mediated mainly via proinflammatory immune cells, plays a key role in causing wounds to become chronic. A characteristic feature of chronic inflammation is the increased presence of proinflammatory cells including neutrophils, macrophages, and T cells for an extended period (3). In this context, proinflammatory T cells can worsen the inflammatory milieu through production of proinflammatory cytokines like IFNγ and TNFα. This creates a feed-forward loop that recruits more proinflammatory neutrophils and macrophages.

T cells become activated and initiate effector responses following crosslinking of the antigen-specific TCR/CD3 complex and costimulatory receptors like CD28 by corresponding MHC bound antigenic peptides and ligands for costimulatory receptors on professional antigen presenting cells (APCs). Activated T cells are primarily identifiable through the upregulation of specific activation markers (e.g. CD69 and CD25), T cell proliferation, and cytokine production (4). Additionally, under proinflammatory conditions direct interaction between keratinocytes and T cells triggers T cell activation (5). Thus, controlling excessive inflammatory activities of T cells can provide an effective treatment strategy in chronic inflammatory skin conditions (6).

Relying on the use of natural products as treatment options is increasing due to their easy accessibility, cost-effectiveness, and usually relatively fewer side effects as compared to conventional drugs (7, 8). *Chamomilla recutita* L., commonly termed Chamomile, is a medicinal plant traditionally used for treating skin irritations, wounds, ulcers, and gastrointestinal disorders (9, 10). At least 120 metabolites have been identified in chamomile extracts, including 36 flavonoids and 28 terpenoids with apigenin (flavonoid), bisabolol (terpenoid), and chamazulene (terpenoid) as the principal bioactive constituents (pure compounds). Several studies have shown anti-inflammatory, anti-bacterial, anti-oxidative properties of chamomile extracts or the pure compounds (11–17). Immunomodulatory effects have been demonstrated in the murine system, specifically on murine macrophages. A few studies have also shown an immunomodulatory potential in the human system specifically on macrophages (16–18). These studies employed single extracts derived from specific plant parts or solvent systems. A comparative analysis among differently prepared extracts and the corresponding pure compounds has been lacking. Furthermore, the ability of these products to directly modulate primary human T cells remains unclear.

Thus, the aim of this study was to comparatively investigate the immunomodulatory potentials of five differentially manufactured chamomile extracts namely, aqueous fermented total extract (CT), aqueous fermented root extract (CR), ethanolic flower extract (CF), ethanolic root extract (CRE), and ethanolic mother tincture (CU) and three characteristic pure compounds normally considered as active compounds in chamomile, namely apigenin (Ap), chamazulene (Cz), and α-bisabolol (Bis) on primary human T cells. Taken together, our results showed an anti-inflammatory effect of chamomile-derived extracts and pure compounds on primary human T cells. However, differential effects were found for the differently prepared extracts.

## Materials and methods

### Materials

Cell culture reagents used in this study were: RPMI-1640 medium (Gibco), L-Glutamine (Gibco), fetal calf serum (FCS) (Pan-Biotech). Additional materials used were: paraformaldehyde (PFA) (Sigma-Aldrich), phosphate buffered saline (PBS) (Sigma-Aldrich), saponin (Sigma-Aldrich), dimethyl sulfoxide (DMSO) (Sigma-Aldrich), ethanol (Honeywell and Merck), ethylene-diamine-tetra-acetic acid (EDTA) (Honeywell), water (HPLC grade, VWR International), acetonitrile (HPLC grade, VWR International), formic acid (98-100%, Merck), stromal cell- derived factor 1alpha (SDF-1α) (R&D Systems), FicoLite-H (Linaris), GolgiStop™ (BD Bioscience), albumin fraction V (Carl Roth), recombinant human IL2 (Peprotech), HEPES (Carl Roth), NaCl (Sigma-Aldrich), CaCl_2_ (Merck). Unlabeled antibodies used were: anti-CD3 (OKT3, in-house, 20 ng/mL), anti-CD28 (555725, BD Bioscience, 75 ng/mL or 5 μg/mL), anti-mouse IgG+IgM (H+L) (115005068, Dianova, 7 μg/mL), Labelled antibodies were specific for the following antigens: CD3-FITC (344804, BioLegend, 1:50), CD3-BV421 (317344, BioLegend, 1:50), CD8-APC-Cy7 (344714, BioLegend, 1:1000), CD25-APC (356110, BioLegend, 1:25), CD69-FITC (310904, BioLegend, 1:25), AnnexinV-PE (640947, BioLegend, 1:20) CD107α (Lamp1)-PE-Cy7 (328618, BD Bioscience, 1:100) GZMB-FITC (560211, BD Bioscience, 1:10), Fluorescent dyes used were: 7-Aminoactinomycin D (7AAD) (420404, BioLegend, 1:100), CellROX™ Green Reagent (C10444,Thermo-Fisher Scientific, 5μM), carboxyfluorescein-diacetate-succinimidyl- ester (CFDA,SE) (C1157, Thermo-Fisher Scientific, 1 μM), eFlour^®^ 670 (65084085 eBioscience™, 62.5 nM).

### Plant extracts and pure compounds

Five differentially prepared *Chamomilla recutita* extracts were provided by WALA Heilmittel GmbH (Aqueous total fermented- CT and aqueous root fermented- CR), Weleda AG (86% ethanolic flower- CF, and 30% ethanolic root- CRE) and DHU-Arzneimittel GmbH & Co. KG (63% ethanolic mother tincture- CU). Both the aqueous fermented extracts were manufactured following the German Homeopathic Pharmacopoeia (GHP 33c), while the ethanolic extracts were produced according to European Pharmacopoeia (Ph.Eur.) 1.1.3 (GHP 2a) (flower), 1.2.12 (GHP 19f) (root), and 1.1.5 (GHP 3a) (mother tincture). Comminuted fresh whole plant or fresh flowers or fresh roots were used to manufacture the extracts. The aqueous extracts were subjected to 7-days fermentation followed by 6-months maturation at 15°C. The ethanolic extracts were pressed and filtered at the end of the extraction process. Apigenin (HPLC, >95% purity) was purchased from Toronto Research Chemicals, while chamazulene (GC, >95% purity) and α-Bisabolol (GC, >93% purity) were purchased from Sigma-Aldrich. All pure compounds were dissolved in DMSO at the indicated concentrations. A maximum threshold of 0.1% was set for the final solvent concentrations in the treatment conditions.

### LC-MS/MS analysis for plant extracts characterization

First, two batches of each chamomile extract were analyzed diluted to approximately 5 mg dw/mL in their respective solvents to reach a comparable normative level (NL) in measurements. All 10 dilutions were ultrasonicated for 2 min, centrifuged at 13000 rpm for 10 min, and transferred to GC-vials. LC-MS/MS analysis was performed on a Finnigan LCQ-Duo ion trap mass spectrometer with an electrospray ionization (ESI) source (ThermoQuest) coupled to an Accela HPLC system (MS pump plus, autosampler, and PDA detector plus) (Thermo-Scientific) with an EC 150/3 Nucleodur 100-3 C18ec column (Macherey-Nagel). A gradient of water and acetonitrile (ACN) with 0.1% formic acid each, was first applied from 5 to 30% ACN in 60 min and to 95% ACN in the next 60 min at 30°C, at 0.5 mL/min flow rate. The injection volume was 20 µl. All samples were measured in ESI+ and ESI- mode. The MS was operated with a capillary voltage of 10 V (ESI+) or -10 V (ESI-), source temperature of 240°C, and high purity nitrogen as a sheath and auxiliary gas at a flow rate of 80 and 40 (arbitrary units), respectively. The ions were detected in a mass range of 50–2000 m/z. A collision energy of 35% was used in MS2 for fragmentation. Data acquisitions and analyzes were carried out by Xcalibur™ 2.0.7 software (Thermo Scientific). The batches of each extract showed an identical composition; therefore, only one batch of each extract was used for all further investigations. The chosen batch samples from the five different chamomile extracts exhibited concentrations between 2.32 and 8.34 mg dw/mL. The selected data were preprocessed in Python because of the big volume. The results were then edited manually. After thorough review, mostly the ESI- results were used for further evaluation. Tentative identification of compounds was done based on the given literature and own calculations on base of the fragmentation patterns.

### Isolation of peripheral blood T cells, culture, and treatment of cells

Peripheral blood mononuclear cells (PBMCs) were isolated from whole blood of healthy donors, using FicoLite-H density-gradient centrifugation. Peripheral blood T cells (PBTs) were then isolated from PBMCs using human Pan T cell isolation kit (Miltenyi), according to the manufacturer’s instructions. The untouched resting PBTs were cultured in complete RPMI medium (RPMI + 10% FCS + 2mM glutamine) at 3 x 10^6^ cells/mL at 37°C, 5% CO_2_. The PBTs were then left untreated or treated with different chamomile extracts, pure compounds, or solvent controls for 1 h at 37°C, 5% CO_2_. The respective doses are given in the figures. This study was approved by the Ethics Committee (S-269/2015). P815 mastocytoma cells (from Watzl, 2013) were cultured in complete RPMI medium at 37°C, 5% CO_2_ and passaged every 2-3 days.

### T cell costimulation

Resting PBTs were kept untreated or treated as described above. Then, they were seeded into well-plates left uncoated (unstimulated) or pre-coated first with 7μg/mL goat-anti-mouse IgG+IgM antibodies, then 20 ng/mL anti-CD3 (OKT3) and 0.075 or 5 μg/mL anti-CD28 antibodies (costimulated samples), spun down at 395 g, 1 min, and costimulated at 37°C, 5% CO_2_ for indicated time points.

### Cell viability assay

Untreated or treated PBTs were kept unstimulated or costimulated for 24 or 72 h as described above. Next, the cells were spun down (395g, 1 min), resuspended in 50 μL of FACS Wash (FW) buffer containing 7AAD dye alone or in combination with additional surface markers, and incubated for 20 min at RT in the dark on a shaker. Then, the cells were washed with FW twice (395 g, 1 min), resuspended in 80-100 μL of FW in FACS tubes and measured at the flow cytometer (LSRII, BD Bioscience). The data were analyzed using FlowJo software. Living cells were gated on 7AAD^-^ population.

### Intracellular ROS measurement

Resting PBTs were kept untreated or treated as described above. The cells were then incubated with or without a reactive oxygen species (ROS) inducer, tertiary-butyl hydroperoxide (TBHP, 200 µM), for 30 min at 37°C, 5% CO_2_. Next, the cells were stained with intracellular ROS sensor dye, CellROX™ Green (5 μM) for 30 min at 37°C, 5% CO_2_. Finally, the cells were washed with PBS twice (395 g, 1 min), and measured immediately at the flow cytometer (LSRII). Data were analyzed using FlowJo software.

### T cell migration assay

Resting PBTs were kept untreated or treated as described above. Next, the lower chamber of a transwell^®^ plate (3 μm pore size) was filled with 200 μL of complete RPMI medium with or without the chemoattractant SDF-1α (100 ng/mL) per sample, followed by loading of 5 x 10^4^ T cells (in 75 μL) onto the upper chamber. The cells were incubated for 90 min at 37°C, 5% CO_2_. Afterwards, the medium with migrated T cells from the lower chamber was carefully transferred to fresh 5 mL FACS tubes, mixed with an equal number of beads per sample, and quantified at the flow cytometer (LSRII).

### T cell surface activation markers analysis

After 24 h costimulation, the untreated/treated T cells (2 x 10^5^ cells/sample) were spun down at 395 g, 1 min, the supernatants were carefully discarded and the cells were resuspended in 50 μL of FACS Wash (FW) buffer (PBS w/o Ca^2+^, Mg^2+^ +1% BSA + 0.7% NaN_3_), containing 7AAD, and antibodies against activation markers CD25 (APC) and CD69 (FITC). The cells were incubated for 20 min at RT in the dark on a shaker. Afterwards, the cells were washed with FW twice (395 g, 1 min), resuspended in 80-100 μL of FW in FACS tubes and measured at the flow cytometer (LSRII). The data expressed as % positive cells and geometric-Mean Fluorescence Intensity (MFI) were analyzed using FlowJo software. Only experiments with statistically significant differences between the unstimulated and costimulated (untreated) samples and non-toxic doses of tested substances were considered for evaluation (**Supplementary Figure 1**).

### T cell proliferation analysis

Resting PBTs (1.5 x 10^5^ cells/sample) were washed twice with PBS (395 g, 6 min), and labelled with the proliferation dye CFDA,SE (1 μM) in PBS (1 x 10^6^ cells/100 µL staining solution) for 15 min at 37°C, 5% CO_2_. Then, the cells were washed twice with prewarmed complete RPMI medium (395 g, 6 min), and adjusted to 0.75 x 10^6^ cells/mL. CFDA,SE dye permeates into the cells, gets cleaved by intracellular esterases, releasing the fluorescent CFSE, that covalently binds to the intracellular amines. The CFSE stained cells underwent treatment and costimulation as described above. After 72 h of costimulation, the cells were spun down (395 g, 1 min), and stained with 7AAD and measured at the LSRII as described above. Division index (DI), depicting the frequency of each cell division per sample according to CFSE dilution, was calculated using FlowJo Software.

### nCounter^®^ gene expression profiling

The nCounter^®^ Human Immunology V2 panel (nanoString Technologies), comprising 579 genes linked to immunological processes/pathways, and 15 internal reference genes for normalization, was used for gene expression profiling. Briefly, resting PBTs (1 x 10^6^ cells/sample) were subjected to 1 h pre-treatment and 4 h costimulation as described above. Next, total RNA was isolated from each sample using Direct-zol RNA miniprep kit (Zymo Research), following the manufacturer’s instructions. RNA purity, yield and integrity were assessed on a Nanodrop^®^ 2000c spectrophotometer (Thermo Fisher Scientific) and Bioanalyzer (Agilent Technologies). Per sample, 25 ng of total RNA (5 µL) was mixed with nCounter^®^ Reporter Codeset (3 µL), Capture Probeset (2 µL) in hybridization buffer (5 µL) and hybridized for 20 h at 65°C. Afterwards, the reactions were cooled down to 4°C, the samples were purified and immobilized on a cartridge, and measured on the nCounter^®^ SPRINT Profiler (nanoString Technologies). Data were analyzed using nSolver™ 4.0 (nanoString Technologies), NormFinder (v0.953, MS Excel add-in) and Graphpad Prism v9.

### Ingenuity pathway analysis

The log2 transformed expression ratios and the p-values of the differentially expressed genes from the nCounter analysis, were imported into the Ingenuity pathway analysis software (Qiagen) (19). A core analysis was run with a p-value threshold of 0.05. Bubble charts depicting the enriched canonical pathways are presented.

### Intracellular cytokines measurement

Resting PBTs (2 x 10^5^ cells/sample) were kept untreated or pre-treated and costimulated for 24 h or 48 h or 96 h as described above. In the final 4 h, Monensin (3 µM) was added to each sample to retain cytokines intracellularly. Then, the cells were washed with PBS twice (395 g, 1 min), surface stained for anti-CD8 in FW (50 μL per sample) for 20 min at RT (dark, shaker). The cells were washed twice with FW (395 g, 1 min), fixed with 1.5% PFA (100 μL/sample) for 10 min at RT (dark, shaker), and permeabilized with FW Saponin (FWS) buffer (FW + 0.1% Saponin) (100 μL/sample) for 15 min at RT (dark, shaker). The cells were then intracellularly stained with anti-IL2 (APC), anti-IFNγ, anti-TNFα, or anti-GZMB (FITC) in FWS (50 μL per sample) for 20 min at RT (dark, shaker). Finally, the cells were washed twice with FWS (395 g, 1 min), resuspended in 80-100 μL of FW and measured at the flow cytometer (LSRII). The data were analyzed using FlowJo software.

### Extracellular cytokines measurement

Resting PBTs (2 x 10^5^ cells/sample) were kept untreated or pre-treated and costimulated for 2 days as described above. Following centrifugation (395 g, 1 min), supernatants from each sample were carefully collected in fresh 1.5 mL Eppendorf tubes and stored at -80°C until measurement. The concentrations of 18 different Th and/or CTL associated cytokines (IL2, IL4, IL5, IL6, IL9, IL10, IL13, IL17A, IL17F, IL22, IFNγ, TNFα, sFas, sFasL, GZMA, GZMB, Perforin, Granulysin were simultaneously detected in the supernatants using LEGENDplex™ Human Th Mix-Match and CD8/NK panel kits (BioLegend) following manufacturer’s instructions. Briefly, 10 μL of test sample or standard were mixed with equal volumes of 1x assay buffer and capture beads in a U-bottom 96-well plate and incubated at RT for 2 h on a shaker in dark. Next, the plate was washed with 200 μL of 1x Wash buffer (300g, 5 min) and 10 μL of Detection antibodies were added to each well and incubated at RT for 1 h on a shaker in dark. Then, 10 μL of Streptavidin-PE beads were directly added to each well and incubated for 30 min at RT on a shaker in dark. Finally, the plate was washed twice with 200 μL of 1x Wash buffer (300g, 5 min). All samples were resuspended in 60 μL of 1x assay buffer and measured at the flow cytometer (BD™ Symphony A3). Data were analyzed using a cloud based LEGENDplex™ Data Analysis Software Suite from Qognit.

### T cell cytotoxicity assay

Resting PBTs (2 x 10^5^ cells/sample) were kept untouched or costimulated as described above for 24 h. The cells were expanded until day 7 in fresh medium containing recombinant human IL2 (40U/mL) every 2 days to generate effector cytotoxic T cells (CTLs). On day 8, the CTLs (1 x 10^6^ cells/mL) were left untreated or treated with indicated concentration of apigenin or solvent control (DMSO) for 1 h, Meanwhile, the target P815 mastocytoma cells were first labelled with eF670 dye in PBS for 15 min at 37°C, 5% CO_2_. After washing twice with complete RPMI medium (395 g, 6 min), P815 cells were kept in complete medium with or without OKT3 (45 ng/mL) for 15 min at 37°C, 5% CO_2_. Just before co-culture, anti-CD107a (PE-Cy7) was added to the CTLs to trap CD107a during upregulation on the surface, and finally combined with P815 at 6:1 (CTL:P815) in a U-bottom 96-well plate for 2 h at 37°C, 5% CO_2_. Afterwards, the cells were washed in 1x Annexin Binding Buffer (ABB) (395 g, 1 min), stained with annexin-V (PE) in 50 μL ABB (per sample) for 20 min at RT (dark, shaker). The cells were washed with 1x ABB twice (395 g, 1 min), followed by surface markers staining, measurement at LSRII and data analysis using FlowJo software.

### Statistical analysis

Statistical analyses were done on GraphPad Prism 9. Two-tailed Student *t* test, One-Way or Two-Way ANOVA were used to compare two or more than two groups respectively. Data are expressed as Mean±Standard Error of Mean (SEM). p-value <0.05 was considered statistically significant. * = p-value < 0.05, ** = p-value < 0.01, *** = p-value < 0.001, **** = p-value < 0.0001.

## Results

### Chamomile extracts show a high heterogeneity in the LC-MS/MS analysis

**Figure 1:**
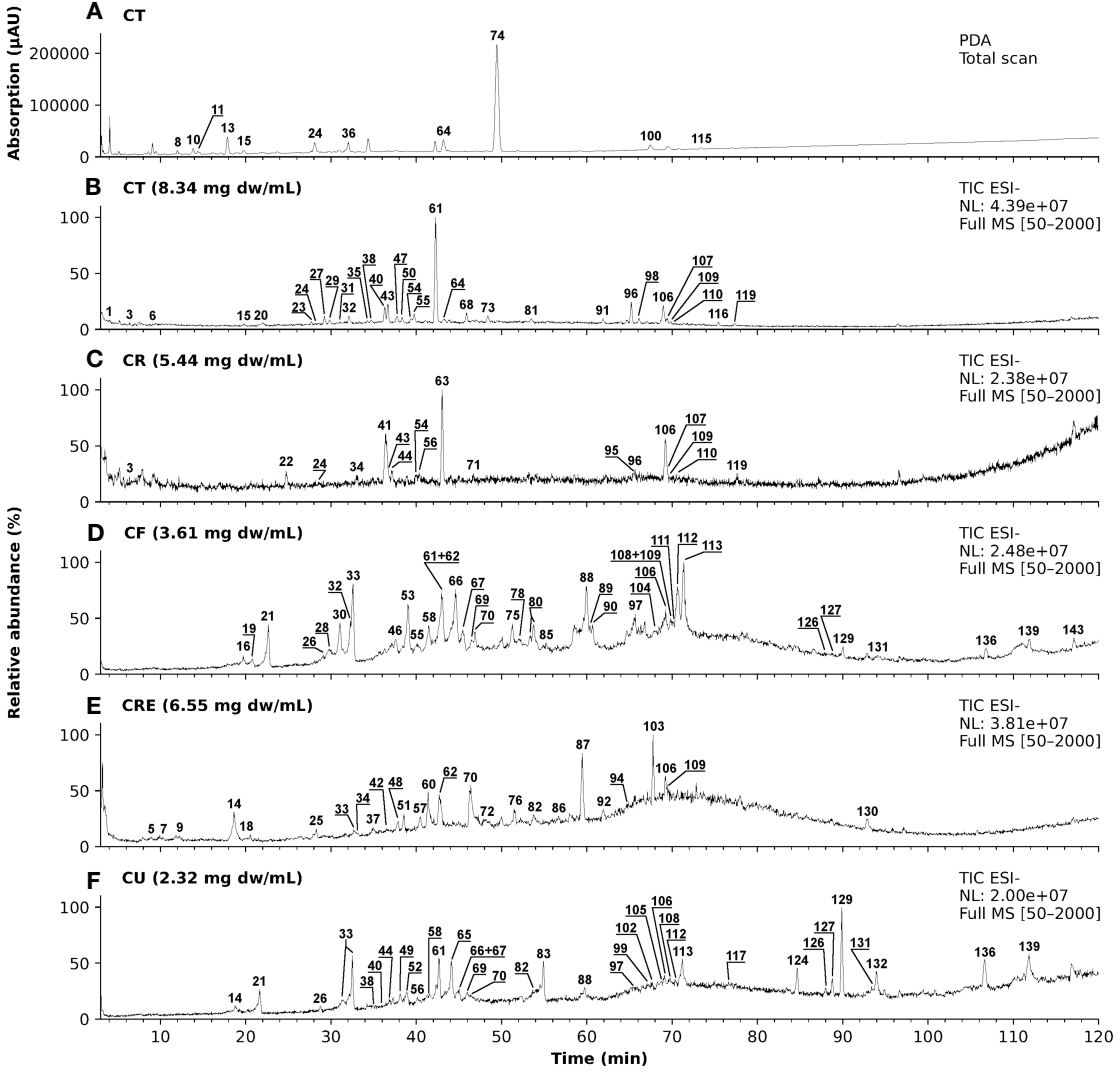
LC-MS/MS chromatograms of five different chamomile extracts with annotated peaks. **(A)** Photodiode array (PDA) chromatogram (λ = 100 - 600 nm) of aq. total. Ferm extract (CT) and **(B-F)** total ion chromatograms (TIC) in ESI- mode of the five tested chamomile extracts): **(B)** aq. total ferm (CT), **(C)** aq. root ferm (CR), **(D)** ethanolic flower (CF), **(E)** ethanolic root (CRE), and **(F)** ethanolic mother tincture (CU).

Extracts of pharmaceutical plants contain several secondary metabolites and phytochemicals that influence the bioactivity. Depending on the plant parts and solvents used for extraction, there can be a high variability in the constituent phytochemicals. Thus, an LC-MS/MS analysis was performed to characterize the metabolites present in the chamomile extracts used in this study. The photodiode array (PDA) chromatogram of aq. total ferm (CT) extract, as an example, and the total ion chromatograms (TICs) with annotated peaks of all five examined extracts (ESI - mode) are shown in **Figure 1**. In **Table 1**, a comparative overview of the tentatively identified metabolites in the different extracts is given. A comprehensive list of all identified and unidentified peaks is included in the supplementary data (**Supplementary Table 1**). Among the identified metabolites, mono- and di-caffeoylquinic acid derivatives occurred in several of the extracts. Phenolic acid like ferulic acid hexosides, and flavonoids like apigenin/luteolin derivatives were detected mainly in the ethanolic flower extract (CF), and ethanolic mother tincture (CU). Interestingly, a cirsiliol derivative was detected in all five extracts (peak 106). However, chamazulene or α-bisabolol, two known pure compounds associated with chamomile oil extracts were not detected in any of the extracts investigated here, neither in pure form nor as derivatives, because of their lipophilic nature. A significant number of peaks remain unidentified due to lack of information in the literature. While different batches of the same extract were homogenous as expected (data not shown), the phytochemical compositions of the five extracts were highly heterogenous which could indicate different bioactivities.

**Table 1:** List of tentatively identified metabolites in the examined chamomile extracts from LC-MS/MS analysis.

Peak No.	Retention time, t_R_ [min]*	ESI mode	m/z		Tentative identification	Occurrence in *C. recutita* extracts (x)				
			Precursor ion	Main product ion		CT	CR	CF	CRE	CU
5	9.0	–	315.8	153.1	Protocatechuic acid *O*-hexoside (20)				x	
6	9.2	–	153.1	109.2	Protocatechuic acid (21, 22)	x	x			
9	12.2	–	353.1	191.2	Caffeoylquinic acid derivative (21, 23, 24)				x	
14	18.6 – 18.7	–	353.1	191.2	Caffeoylquinic acid derivative (21, 23, 24)				x	x
16	19.7	–	353.1	191.2	Caffeoylquinic acid derivative (21, 23, 24)			x		
19	20.8	–	355.1	193.0	Ferulic acid hexoside (21, 25)			x		
21	21.7	–	355.1	193.0	Ferulic acid hexoside (21, 25)			x		x
23	27.7	–	593.3	473.2	Apigenin 6,8-di-*C*-hexoside (26, 27)	x				
24	28.2 – 28.7	+	163.1	107.1	Umbelliferone (28)	x	x			
28	29.7	–	355.0	193.0	Ferulic acid hexoside (21, 25)			x		
30	31.1	–	355.0	193.0	Ferulic acid hexoside (21, 25)			x		
32	32.1 – 32.3	–	355.1	193.0	Ferulic acid hexoside (21, 25)	x		x		
33	32.5 – 32.7	–	355.1	193.0	Ferulic acid hexoside (21, 25)			x	x	x
34	33.0	+	223.2	208.0	Isofraxidin (29)		x		x	
46	37.6	–	493.2	331.2	Petuletin glucoside (26)			x		
49	38.1	–	593.3	285.2	Luteolin diglycoside (27)					x
52	38.9	–	447.3	285.2	Luteolin hexoside (27)					x
53	39.1	–	493.2	331.2	Petuletin glucoside (26)			x		
57	40.4	–	187.2	125.2	Azelaic acid (30)				x	
60	41.4	–	515.3	353.1	Dicaffeoylquinic acid derivative (24)				x	
61	42.3 – 42.7	–	431.3	269.3	Apigenin glucoside derivative (31)	x				x
62	42.7 – 43.0	–	515.1	353.1	Dicaffeoylquinic acid derivative (24)			x	x	
64	43.3	–	193.1	149.1	Ferulic acid	x				
65	44.1	–	431.3	269.3	Apigenin glucoside derivative (31)					x
66	44.6 – 45.0	–	431.3	269.2	Apigenin glucoside derivative (31)			x		x
67	45.5	–	477.2	315.2	Isorhamnetin glucoside (26)			x		
69	46.0 – 46.4	+	463.0	301.2	Chrysoeriol-7-*O*-glycoside (32)					x
70	46.3 – 47.0	–	515.2	353.1	Dicaffeoylquinic acid derivative (24)			x	x	x
72	48.1	–	515.2	353.1	Dicaffeoylquinic acid derivative (24)				x	
75	51.3	–	655.2	331.2	Pentahydroxymethoxyflavone caffeoylglucoside (26)			x		
78	52.1	–	473.3	269.3	Apigenin acetyl-glucoside derivative (31)			x		
80	53.8	–	473.3	269.3	Apigenin acetyl-glucoside derivative (31)			x		
88	59.7 – 60.0	–	473.3	269.3	Apigenin acetyl-glucoside derivative (31)			x		x
89	60.3	–	473.3	269.3	Apigenin acetyl-glucoside derivative (31)			x		
90	60.7	–	473.3	269.3	Apigenin acetyl-glucoside derivative (31)			x		
97	65.3 – 65.5	–	269.3	269.2	Apigenin			x		x
99	67.0	–	299.2	284.2	Hispidulin (31)					x
102	67.7	–	785.4	665.3	Tetra-*cis*/*trans*-coumaroyl polyamine derivative (32–34)					x
104	67.9	–	785.5	665.3	Tetra-*cis*/*trans*-coumaroyl polyamine derivative (32–34)			x		
105	68.8	–	785.4	665.3	Tetra-cis/trans-coumaroyl polyamine derivative (32–34)					x
106	69.0 – 69.2	–	329.3	229.4	Cirsiliol derivative (35, 36)	x	x	x	x	x
107	69.3 – 69.5	–	329.3	229.2	Cirsiliol derivative (35, 36)	x	x			
108	69.7 – 70.0	–	785.4	665.3	Tetra-*cis*/*trans*-coumaroyl polyamine derivative (32–34)			x		x
109	69.4 – 69.9	–	329.3	229.3	Cirsiliol derivative (35, 36)	x	x		x	
111	70.2	–	785.5	665.4	Tetra-*cis*/*trans*-coumaroyl polyamine derivative (32–34)			x		
112	70.3 – 70.7	–	785.4	665.3	Tetra-*cis*/*trans*-coumaroyl polyamine derivative (32–34)			x		x
113	71.2 – 71.3	–	785.4	665.3	Tetra-*cis*/*trans*-coumaroyl polyamine derivative (32–34)			x		x

CT: aq. total ferm, CR: aq. root ferm, CF: ethanolic flower, CRE: ethanolic root, CU: mother tincture. *: tR is based on the MS data. Delay time 0.06 – 0.09 min; t0 was not subtracted (1.85 min in PDA).

### Chamomile extracts and pure compounds differentially affect intracellular ROS levels in human T cells under oxidative stress conditions

**Figure 2:**
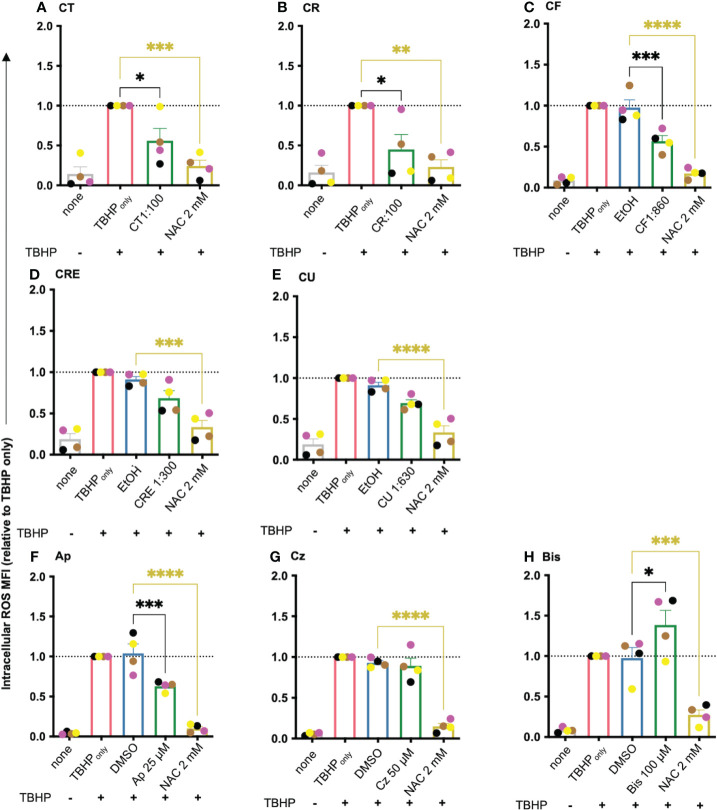
Chamomile extracts and pure compounds differentially influence intracellular ROS level in T cells. PBTs were kept untreated or treated with indicated doses of extracts/pure compounds/solvent controls for 1 h. Oxidative stress was induced by TBHP. Intracellular ROS levels were measured using CellROX™ Green. Extracts: **(A)** CT, **(B)** CR, **(C)** CF, **(D)** CRE, **(E)** CU. Pure compounds **(F)** Ap, **(G)** Cz, **(H)** Bis. N=4. Each color-coded symbol represents an individual donor. Grey bars = untreated (none), red bars = TBHP control, blue bars = solvent controls, green bars = extracts or pure compounds, yellow bars = positive control NAC. Data were normalized to “TBHP only” samples. Statistical analysis using One-way ANOVA with Tukey’s post-hoc test was performed and the results are expressed as Mean ± SEM. * = p-value < 0.05, ** = p-value < 0.01, *** = p-value < 0.001, **** = p-value < 0.0001.

T cell activation and downstream processes are influenced by the redox micromilieu. While at low concentrations, ROS act as signaling molecules, at higher concentrations, they induce oxidative stress in T cells. Chamomile extracts and characteristic pure chamomile compounds are known to have antioxidative effects (37–41). Whether they also have antioxidative effects in T cells remained unknown. Therefore, after having identified non-toxic doses of the extracts and pure compounds at various time points by a viability assay [**Supplementary Figure 1** (24 h), **Supplementary Figure 2** (72 h)], their effects on the intracellular ROS levels in primary human T cells were analyzed using the CellROX™ Green ROS sensor. To this end, first TBHP (200 μM) was added to the T cells to induce oxidative stress, followed by incubation with CellROX™ Green (5 μM), which becomes highly fluorescent under oxidative condition. The changes in the MFI of the intracellular CellROX™ Green fluorescence per cell were detected by flow cytometry. The antioxidant N-acetyl cysteine (NAC, 2 mM) was used as a positive control in these experiments. Chamomile aq. total ferm (CT, 1:100), aq. root ferm (CR, 1:100), and ethanolic flower (CF, 1:860) extracts significantly decreased the MFI of intracellular ROS, while the ethanolic root extract (CRE, 1:300) and the mother tincture (1:630) showed only a tendency to decrease the intracellular ROS (**Figures 2A-E**) (CRE p-value = 0.1392, CU p-value = 0.668). The pure compound apigenin (Ap, 25 μM) significantly decreased the MFI, chamazulene (Cz, 50 μM) showed no prominent effect, and α-bisabolol (Bis, 100 μM) showed an increase in the MFI of intracellular ROS (**Figures 2F-H**). Taken together, different chamomile extracts and pure compounds showed differential effects on intracellular ROS levels in T cells under oxidative stress conditions.

### Chamomile extract CR inhibits T cell migration while the other extracts and pure compounds show no prominent effects

**Figure 3:**
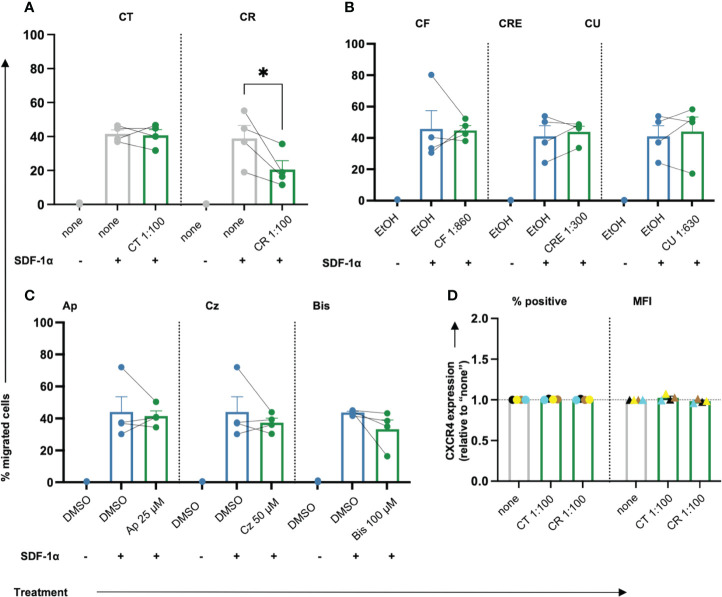
Only chamomile extract CR diminishes SDF-1α induced T cell migration significantly. PBTs were kept untreated (none) or treated with solvent controls (EtOH/DMSO) or different doses of extracts or pure compounds for 1 h and analyzed for the migration capacity using Transwell-insert 96-w-plate and the chemoattractant SDF-1α. The percentage of migrated cells was determined after 1.5 h. Extracts: **(A)** CT, CR, **(B)** CF, CRE, CU. Pure compounds **(C)** Ap, Cz and Bis. **(D)** Surface expression of CXCR4 (normalized to untreated sample “none”) was not affected by CT or CR. N=4. Each dot represents an individual donor [color-coded in **(D)**]. Gray bars/dots = untreated (none) controls. Blue bars/dots = solvent controls (EtOH/DMSO). Green bars/dots = extracts or pure compounds. Statistical analysis was performed using **(A–C)** One-way ANOVA with Šídák’s post-hoc test or **(D)** Two-way ANOVA with Dunnett’s post-hoc test, and the results are expressed as Mean ± SEM. * = p-value <0.01.

T cells migrate in search of cognate antigens, which is crucial for the initial antigen encounter as well as the consecutive effector response during infections and (chronic) inflammation. In low doses, ROS can promote T cell activation and migration, while higher doses of ROS inhibit T cell activation and migration, at least in part due to oxidation of the actin-binding proteins cofilin and L-plastin (42, 43). Given the observed diminishment of intracellular ROS in primary human T cells following treatment with chamomile extracts CT, CR, CF, and pure compound Ap, we next tested the effects of the different extracts and pure compounds on the migration capacity of T cells. SDF-1α, a physiological T cell chemoattractant known to be relevant in chronic inflammation (44), was used to induce migration of T cells during 1.5 h incubation in a transwell^®^ plate. Among the extracts, only the aq. root ferm extract (CR, 1:100) significantly decreased the % migrated T cells while the other extracts showed no prominent influence (**Figures 3A, B**). The pure compounds also showed no prominent effect on T cell migration (**Figure 3C**). Inhibition of T cell migration by CR could be due to interference with the expression of CXCR4, the receptor for SDF-1α. However, CR did not affect the surface expression of CXCR4 (**Figure 3D**). Altogether, only chamomile extract CR inhibited SDF-1α-induced T cell migration while the other extracts/pure compounds showed no significant effects on the migration of primary human T cells.

### Chamomile extracts and pure compounds show differential effects on the expression of activation markers in T cells

**Figure 4:**
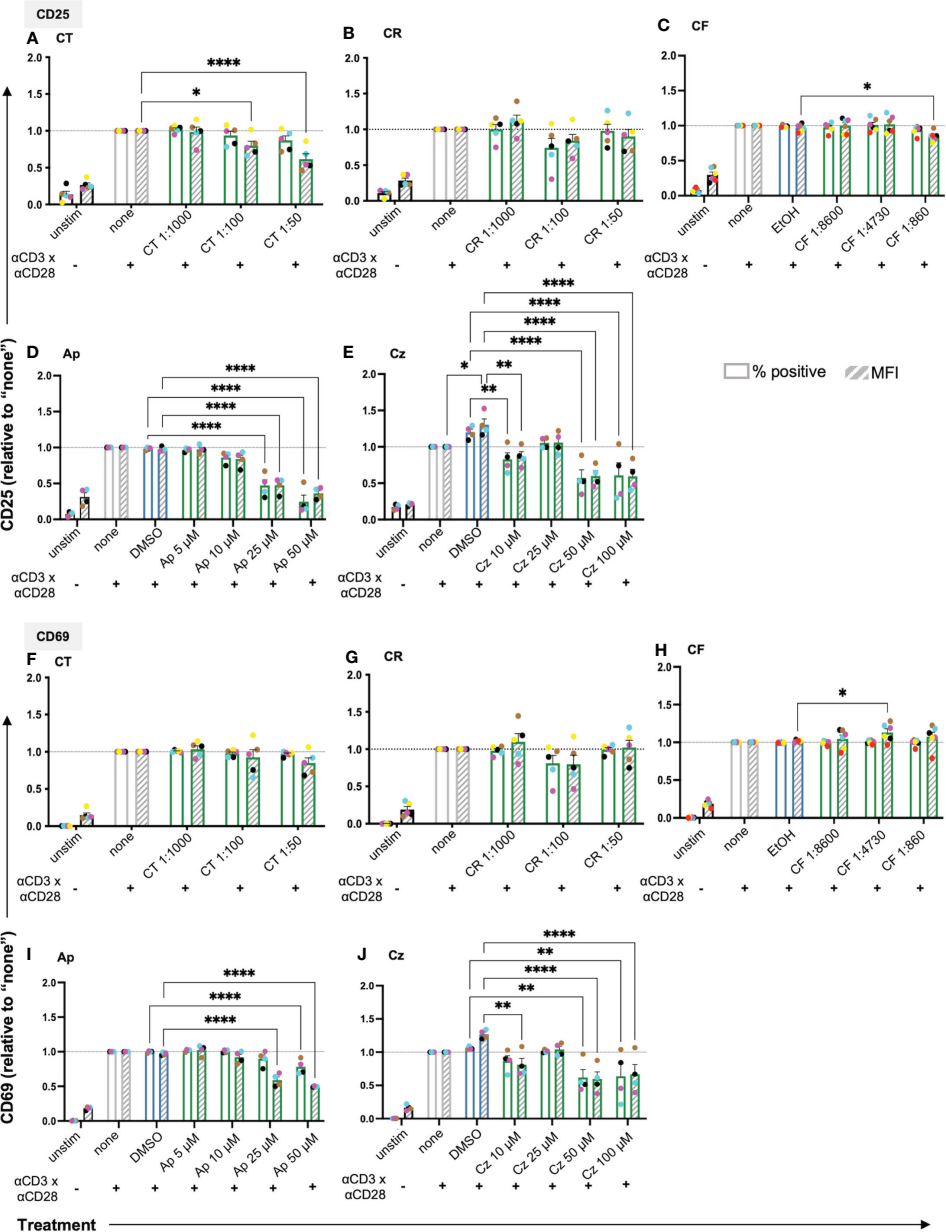
The chamomile extracts and the pure compounds differentially affect the expression of the T cell activation markers CD25 and CD69. PBTs were kept untreated (none) or treated for 1 h and analyzed for the surface expression of **(A-E)** CD25 and **(F-J)** CD69 after anti-CD3/CD28 costimulation for 24 h. Extracts: **(A, F)** CT, **(B, G)** CR and **(C, H)** CF. Pure compounds: **(D, I)** Ap, **(E, J)** Cz. Each colored dot represents an individual donor (N = 4-6). Black bars= unstimulated (unstim), grey bars = untreated controls (none), blue bars = EtOH/DMSO controls, green bars = extracts or pure compounds. (
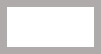
) = % positive (
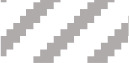
) = geometric Mean Fluorescence Intensity (MFI). Data were normalized to the untreated samples (none). Statistical analysis was performed using Two-way ANOVA with Dunnett’s post-hoc test, comparing against their respective controls (none/EtOH/DMSO) and the results are expressed as Mean ± SEM. *=p-value <0.05, **=p-value <0.01, ****=p-value <0.0001.

Following costimulation through TCR/CD3 and CD28 (CD3xCD28), which mimics antigen recognition on an APC, T cells upregulate surface markers like CD25 (IL2Rα) and CD69 as classical activation markers (**Supplementary Figure 3A**). To check whether the extracts/pure compounds affected T cell activation, surface expression of CD25 and CD69 on the anti-CD3xCD28 costimulated T cells was assessed using flow cytometry. Among the extracts, aq. total ferm (CT) significantly lowered CD25 MFI dose-dependently, aq. root ferm (CR, 1:100), and ethanolic flower (CF,1:860) showed a slight tendency to lower CD25 MFI (**Figures 4A-C**) (CR p-value = 0.3906) whereas ethanolic root (CRE) and ethanolic mother tincture (CU) did not show a prominent effect on CD25 expression (**Supplementary Figure 3B**). Among the pure compounds, Ap and Cz significantly lowered both % CD25 positive cells and its expression level (MFI) (**Figures 4D, E**), whereas Bis showed inconsistent effects on the CD25 expression (**Supplementary Figure 3B**). Evaluating the effect on CD69 expression showed no prominent influence by CT, a small tendency to decrease CD69 by CR (1:100) (p-value = 0.1581), while a significant, but still small increase of CD69 was observed in the presence of CF (1:4730) (**Figures 4F-H**). In contrast, CRE and CU did not show a prominent effect on CD69 expression (**Supplementary Figure 3B**). Ap and Cz significantly lowered CD69 expression (**Figures 4I, J**), but Bis showed inconsistent effects on CD69 expression (**Supplementary Figure 3B**). In sum, chamomile extracts and pure compounds exhibited differential effects on the expression of the activation markers CD25 and CD69 in costimulated T cells, while Ap and Cz decreased the expression of both activation markers.

### Chamomile extracts CT, CR and CF, and pure compounds Ap and Cz inhibit proliferation capacity of T cells

Once activated, the T cells proliferate rapidly (clonal expansion) to bring forth appropriate effector responses. Thus, the effects of the extracts and pure compounds on the T cell proliferation capacity were evaluated using the proliferation tracking dye CFSE. As cells divide, the CFSE splits equally among the daughter cells, characterized as subsequent individual peaks with diminishing fluorescence (**Supplementary Figure 4A**). The Division Index (DI), a parameter indicating the number of divisions undergone by each cell per sample, was used to evaluate the proliferation capacity of the T cells. Only non-toxic doses of extracts/pure compounds were used for subsequent assessment (**Supplementary Figure 2**).

**Figure 5:**
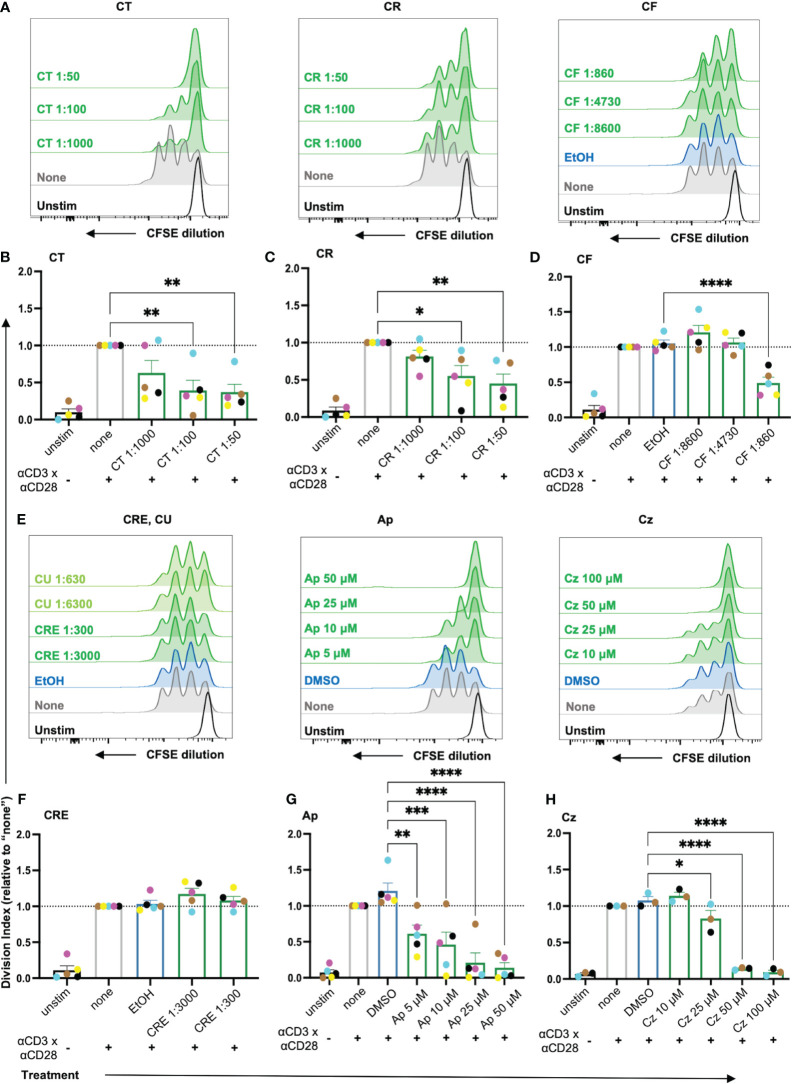
The proliferation of costimulated T cells was significantly reduced by the chamomile extracts CT, CR, CF and the pure compounds Ap and Cz. CFSE-stained PBTs were kept untreated (none) or treated with solvent controls (EtOH/DMSO) or different doses of extracts/pure compounds for 1 h and analyzed for proliferation capacity after 72 h anti-CD3/CD28 costimulation. **(A, E)** Representative histograms, and **(B-D, F-H)** statistical evaluation depicting proliferation of costimulated T cells. Extracts: **(A, B)** CT, **(A, C)** CR, **(A, D)** CF, **(E, F)** CRE. Pure compounds **(E, G)** Ap **(E, H)** Cz. N=3-5. Each colored-dot represents an individual donor. Black bars= unstimulated (unstim), grey bars = untreated controls (none), blue bars = EtOH/DMSO controls, green bars = extracts or pure compounds. Data were normalized to the untreated samples (none). Statistical analysis was performed using One-way ANOVA with Dunnett’s post-hoc test, comparing against their respective controls (none/EtOH/DMSO) and the results are expressed as Mean ± SEM. * = p-value <0.05, ** = p-value <0.01, *** = p-value <0.001, ****=p-value <0.0001.

Among the extracts, aq. total ferm (CT), aq. root ferm (CR) and ethanolic flower (CF) significantly lowered the DI of the CD3xCD28 costimulated cells (**Figures 5A-D**), while ethanolic root (CRE) (**Figures 5E, F**) and ethanolic mother tincture (CU) (**Supplementary Figure 3**) did not affect the DI. Among the pure compounds, Ap and Cz significantly lowered the DI (**Figures 5G, H**), while Bis lowered the DI by trend (**Supplementary Figure 3**). Taken together, chamomile extracts CT, CR and CF, as well as pure compounds Ap and Cz significantly inhibited the proliferation capacity of costimulated T cells.

### Chamomile extracts and pure compounds show differential effects on cytokine production

**Figure 6:**
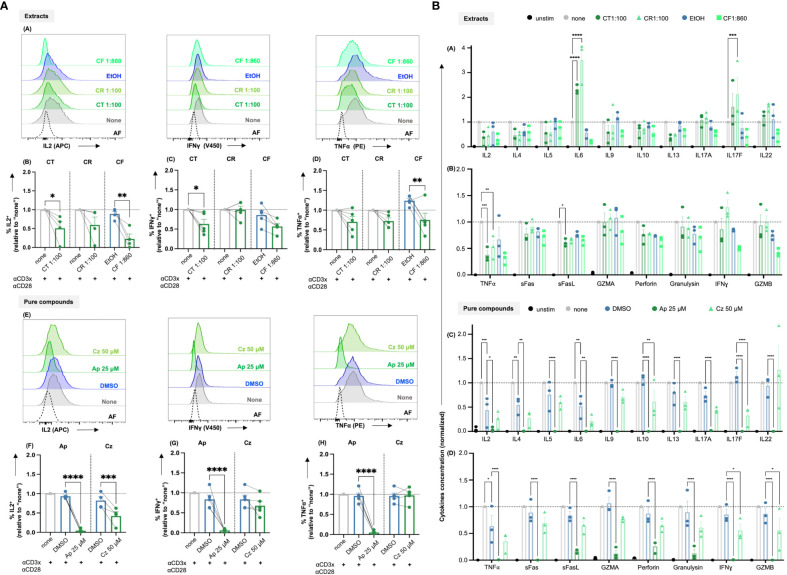
**(A)** Chamomile extracts and pure compounds differentially affect intracellular IL2, IFNγ and TNFα levels. PBTs were kept untreated (none) or treated with solvent controls (EtOH/DMSO) or different extracts or pure compounds at indicated doses for 1 h and costimulated for 48 h. Monensin was added to the samples in the final 4 h of costimulation. Afterwards, intracellular IL2, IFNγ and TNFα levels were measured. (A, E) Representative histograms and (B-D, F-H) Statistical evaluations of %IL2^+^ cells (normalized), %IFNγ^+^ cells (normalized) and %TNFα^+^ cells (normalized). Extracts: (A-D) CT, CR, CF. Pure compounds: (E-H) Ap, Cz. N=4-5. Each dot represents an individual donor. Grey bars/dots = untreated controls (none), blue bars/dots = EtOH/DMSO controls, green bars/dots = extracts or pure compounds. AF = Autofluorescence. Statistical analysis using One-way ANOVA with Šídák’s post-hoc test was performed and the results are expressed as Mean ± SEM. **** = p-value < 0.0001. **(B)** Chamomile extracts and pure compounds show differential effects on cytokine production. PBTs were left unstimulated (unstim) or were kept untreated (none) or treated with different extracts/ pure compounds /vehicles for 1 h and costimulated with anti-CD3/CD28 for 48 h. Supernatants were collected and quantified for cytokine production using LEGENDplex^™^ Human Th mix-match and CD8/NK kits. (A, B) Differential effects of the different extracts as indicated were observed in the levels of 18 Th or CTL related cytokines. (C, D) Apigenin significantly reduced the levels of all 18 cytokines, but Chamazulene showed differential effects. N=3, each dot represents a donor. Data were normalized to the respective controls (none). Statistical analysis was performed using Two-Way ANOVA with Tukey’s post-hoc test and data are expressed as Mean ± SEM. *=p-value <0.05, **=p-value <0.01, ***=p-value <0.001, ****=p-value <0.0001.

To facilitate their proliferation, T cells produce the cytokine IL2, which acts as a growth factor by binding to the upregulated high affinity IL2 receptor (CD25) in an autocrine or paracrine manner. Thus, to check the effects of the promising chamomile extracts and pure compounds on activation-induced IL2 production, an intracellular staining after 48 h of CD3xCD28 costimulation was performed. The intracellular IL2 level was found to be significantly reduced by the extracts aq. total ferm (CT, 1:100) and ethanolic flower (CF, 1:860). The aq. root ferm (CR, 1:100) diminished the intracellular IL-2 content only by trend (p-value = 0.1220), likely due to the observed high donor variation (**Figures 6A**-A, B). The pure compounds Ap (25 μM) and Cz (50 μM) also significantly reduced the intracellular IL2 level (**Figures 6A**-E, F). In conclusion, the diminishment of both, IL2 induction and CD25 expression (by trend or significantly), may explain the inhibitory action of CT, CR, CF, Ap and Cz on T cell proliferation. Note that exogenous supplementation of IL2 could not reverse the inhibitory effects of these extracts and pure compounds on T cell proliferation (**Supplementary Figure 4**).

Additionally, the effects of the extracts and the pure compounds on the induction of major proinflammatory cytokines, namely IFNγ and TNFα, were investigated by intracellular staining after 48 h of CD3xCD28 costimulation. Among the three extracts, the intracellular IFNγ level was lowered by CT (significantly), and CF (by trend due to high donor variation, p-value = 0.0788), but not affected by CR (**Figures 6A**-A, C). For TNFα, CT and CR lowered its level only by trend (CT p-value = 0.1209, CR p-value = 0.1594), which likely results from high donor variation, while CF lowered intracellular TNFα levels significantly (**Figures 6A**-A, D). Also the pure compound Ap decreased IFNγ and TNFα levels significantly, but Cz showed only a tendency to lower IFNγ (likely also due to high donor variation, p-value = 0.5) and did not affect TNFα level (**Figures 6A**-E, G, H). In sum, the extracts and pure compounds differentially inhibited the induction of IFNγ and TNFα.

T cells produce several cytokines to mediate specific effector functions. To get an overview of the influence of the most promising extracts CT, CR, and CF and pure compounds Ap, and Cz, on the different cytokines associated with both CD4^+^ and CD8^+^ T cells, a bead-based multiplex assay (LEGENDplex™) was performed using the supernatants of the CD3xCD28 costimulated T cells after 48 h. The quantification of the 18 cytokines, normalized to respective controls, is summarized in **Figure 6B**. The three extracts showed differential effects on the levels of cytokines. Interestingly, CR and CT increased IL6 levels significantly, CR in addition increased IL17F levels significantly, CT only by trend. In contrast, TNFα was significantly decreased by both CR and CT, while the expression of sFasL was only significantly inhibited by CT. In line with the inhibition of intracellular IL2 (compare **Figure 6A**) and T cell proliferation (compare **Figure 5**), all extracts decreased the IL2 levels in the supernatants at least by trend. Among the pure compounds, Ap significantly decreased the levels of all 18 cytokines, while Cz significantly decreased 4 cytokines, namely IL10, IL17F, IFNγ and GZMB. Of note, the solvent controls EtOH and DMSO were also found to affect the concentrations of some of the cytokines. However, these effects were statistically significant only in case of DMSO (IL2, IL4, and TNFα). Therefore, the effects of the pure compounds dissolved in DMSO were directly compared to the effects of the solvent control DMSO. All in all, the different chamomile extracts and pure compounds differentially affect the cytokine production by costimulated T cells.

### Chamomile extracts and pure compounds inhibit mainly proinflammatory cytokine genes related to activation and differentiation of T cells

**Figure 7:**
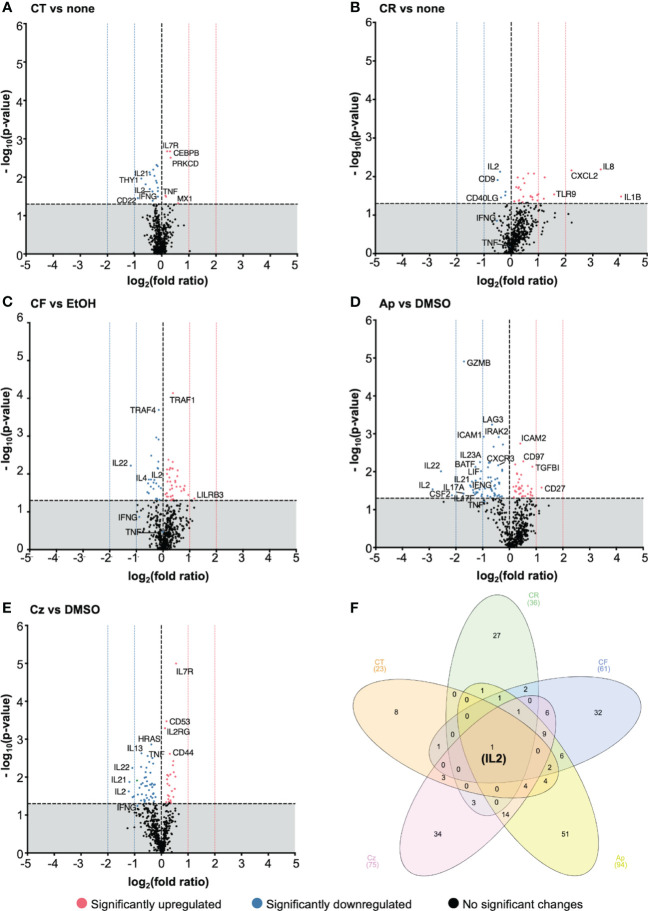
Chamomile extracts and pure compounds differentially regulated gene expression. PBTs were kept untreated or treated 1 h, costimulated for 4 h with plate-bound anti-CD3xCD28 antibodies, and total RNA was isolated and analyzed using nCounter^®^ gene expression profiling. Treated samples were compared to respective controls (none/EtOH/DMSO). The log_2_(fold ratios) and the -log_10_(p-values) are plotted on the x and y axes of the volcano plots respectively. Extracts: **(A)** CT, **(B)** CR, **(C)** CF. Pure compounds: **(D)** Ap, **(E)** Cz. **(F)** A venn diagram showing the number of significantly regulated, overlapping and distinctive genes by the different treatment conditions. N=5 Statistical analysis was performed using pairwise Student’s t-Tests. Horizontal dotted line (y = 1.3) is the significance threshold. Vertical dotted lines indicate the extent of fold ratios. (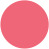) = significantly upregulated genes, (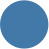) = significantly downregulated genes, (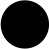) = genes with no significant changes.

To investigate the effects of the promising extracts and pure compounds at a broader molecular level, an nCounter gene expression profiling was performed, using the human immunology panel v2, comprising 579 genes associated with key immunological processes/pathways and 15 internal reference genes. Volcano plots depicting the up and down regulated genes after different treatment conditions are shown in **Figures 7A-E**. A gene with a positive log_2_ fold ratio indicates upregulation (right) or vice-versa downregulation (left). A summary of significant genes from all treatments is given in the supplementary data (**Supplementary Table 2**).

The chamomile aq. total ferm (CT, 1:100) significantly downregulated 17 and upregulated 6 genes, aq. root ferm (CR, 1:100) showed 5 down- and 31 up-regulated genes, and ethanolic flower (CF, 1:860) showed 21 down- and 38 up-regulated genes. The pure compound Ap (25μM) significantly downregulated 58 and upregulated 36 genes. Specifically, top downregulated genes included proinflammatory cytokine genes like *IL2, IL22, CSF2, IL17-A, IL17-F, IL21, IL23A, GZMB* (>(-)1 log_2_ fold ratios). On the other hand, *CD27*, a costimulatory molecule, was significantly upregulated by Ap (>1 log_2_ fold ratio). Cz (50μM) significantly downregulated 49 and upregulated 26 genes. Specifically, *IL2, IL21* and *IL22* were the top downregulated genes (>(-)1 log_2_ fold ratios), whereas *IL7R* was significantly upregulated (but <1 log_2_ fold ratio).

However, interestingly, all the tested extracts and pure compounds significantly downregulated *IL2* transcription (**Figure 7F**). This is in line with the decrease in IL2 production by the promising extracts and pure compounds (**Figures 6A**, **B**). Transcriptional and translational downregulation of IL2, along with CD25 diminishment (by trend or significantly), may explain their inhibitory action on T cell proliferation.

To get an overview of the biological pathways associated with the differentially regulated genes, an Ingenuity Pathway Analysis (IPA) was performed with the significant genes from nCounter run, to directionally predict the enriched canonical pathways. Pathways with activation z-scores >∣2 were considered significantly enriched. These genes were mainly linked to TCR signaling, T cell activation, and differentiation. Th1/Th17 activation, IL17 signaling pathways were among the top pathways predicted to be downregulated in almost all treatment conditions (**Supplementary Figure 5**, **Supplementary Table 3**). Besides, non-T cell specific pathways like macrophage classical activation, pathogen induced cytokine storm signaling pathways were also predicted to be downregulated by the different treatment conditions, except CR treatment. Among the top pathways predicted to be upregulated were erythropoietin signaling, and CDX gastrointestinal cancer signaling pathways. Indication of biases in certain pathways was presumably due to few overlapping genes in the dataset to those in specific pathways. Summarized these data show that the chamomile extracts and pure compounds downregulated several cytokine genes linked with activation and differentiation of costimulated T cells.

### Apigenin lowers granzyme B level and killing capacity of T cells

**Figure 8:**
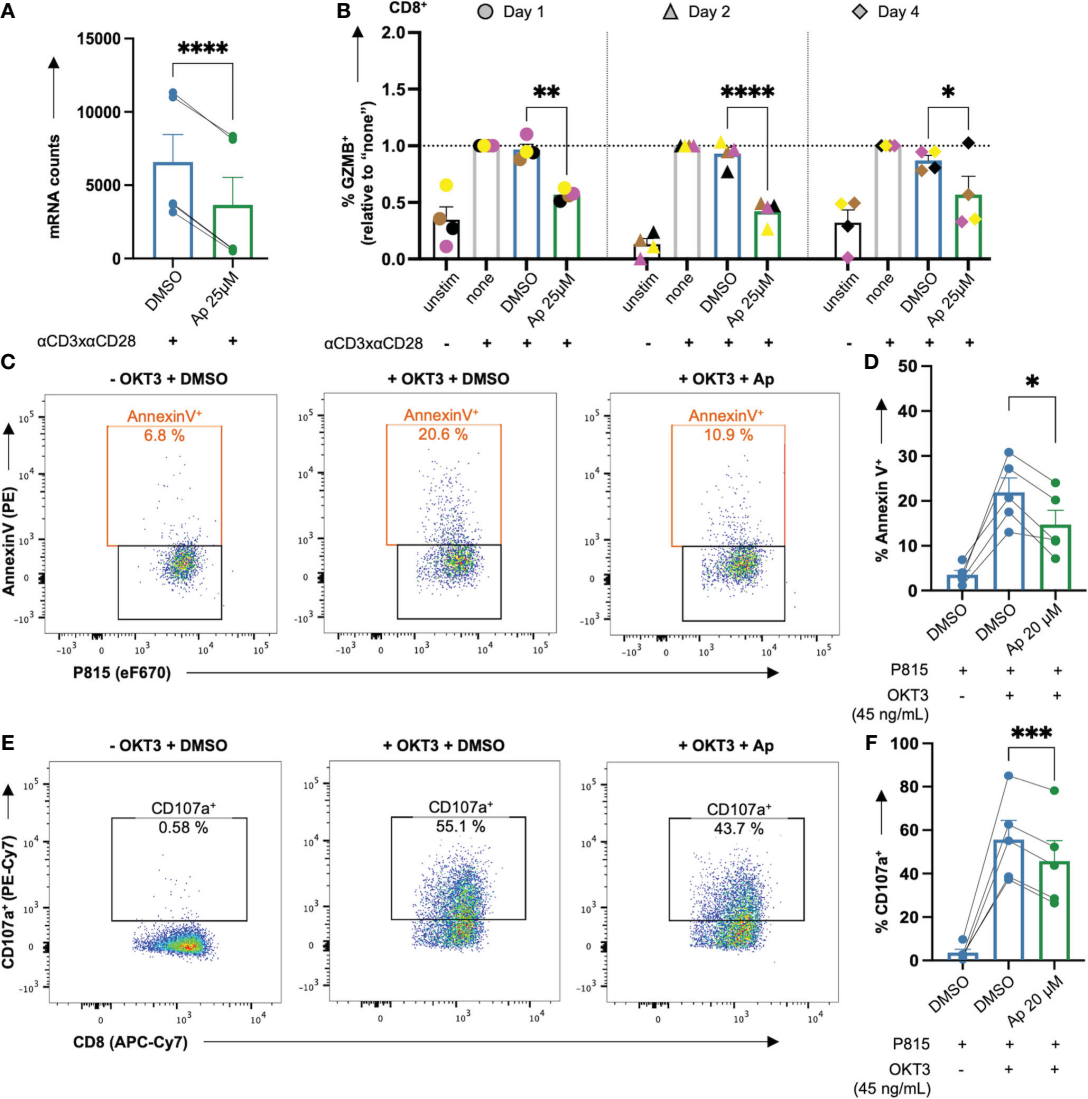
Apigenin lowers GZMB induction in costimulated CD8^+^ T cells and the killing capacity of CTLs. **(A, B)** Untreated or DMSO or apigenin-treated PBTs were costimulated with anti-CD3xCD28 for 1, 2 & 4 days. Monensin (3 μM) was added to the cell culture in the final 4 h costimulation. Afterwards, intracellular GZMB was detected in the costimulated T cells. **(A)** Ap decreased mRNA counts (normalized to DMSO) of GZMB from nCounter analysis, **(B)** Ap decreased the intracellular GZMB protein level at all three time points in CD8^+^ T cells. **(C-F)** PBT were costimulated for 7 days. The resulting effector CTLs were kept untreated or treated with Ap for 1h and co-cultured with the target P815 cells (day 8). **(C, D)** Annexin V staining indicating % apoptotic P815 target cells **(E, F)** Degranulation indicated by % CD107a^+^ effector CTLs. N=4-5. Each color-coded symbol represents an individual donor. Black bars = unstimulated (unstim), grey bars = untreated controls (none), blue bars = DMSO controls, green bars = Ap treated samples. Data for GZMB detection were normalized to respective controls (“none”). Statistical analyses using **(A)** Student’s t-Test, **(D, F)** One-way ANOVA or **(B)** Two-way ANOVA with Dunnett’s post-hoc test were performed and results are expressed as Mean ± SEM. * = p-value < 0.05, ** = p-value < 0.01, *** = p-value < 0.001, **** = p-value < 0.0001.

One of the most prominent effects in the nCounter analysis of gene expression and the LEGENDplex™ cytokine measurement was the highly significant downregulation of granzyme B (GZMB) by apigenin (Ap) (**Figures 6B**, **7D**, **8A**). To investigate, whether Ap inhibits the killing capacity of T cells, first the intracellular GZMB level was assessed. Ap significantly lowered % GZMB^+^ cells in CD8^+^ (**Figure 8B**), and CD4^+^ T cells (**Supplementary Figure 6A**) after 1, 2 and 4 days.

Next, the killing activity of CTLs, which is at least partially mediated by GZMB, was assessed in the presence or absence of Ap, using a CD3 antibody (OKT3) loaded P815 mastocytoma target and effector CTL co-culture system. To this end, CTLs were generated from PBTs after anti-CD3xCD28 costimulation and subsequent expansion with IL2 (40 U/mL) for 7 days, followed by 1 h pretreatment with Ap before co-culture on day 8. Note that in a pre- test, Ap 20 μM showed similar effects as Ap 25 μM, however, with greater viability of CTLs (data not shown). Thus, CTL tests were done with Ap 20μM. Interestingly, CTL-induced apoptosis of P815 cells (increased % AnnexinV^+^) upon OKT3 cross-linkage was indeed halved in case of Ap-treated CTLs (**Figures 8C, D**). Mechanistically, degranulation of CTLs (increased % CD107a^+^) after crosslinking, was significantly decreased with Ap-treated CTLs (**Figures 8E, F**). Note that within these effector CTLs, GZMB was already present before incubation with Ap and these levels did not change during this 1 h preincubation period with Ap (**Supplementary Figure 6B**). In summary, Ap could diminish both the induction of GZMB in primary T cells and the killing capacity of CTLs by lowering their proapoptotic and degranulation capacities.

## Discussion

Chamomile extracts and the associated pure compounds are known to have anti-inflammatory, anti-oxidative, anti-bacterial, and wound healing properties (9, 12, 40, 41). In this study, we have characterized potential immune modulatory effects of chamomile extracts and respective pure compounds of chamomile on primary human T cells. These cells are key regulators of many immune responses. For the first time, at least to our knowledge, a comparative analysis of five differentially prepared chamomile extracts and three selected pure compounds was performed to determine their ability to modulate primary human T cells at a functional and molecular level. Our study demonstrated that various non-toxic chamomile extracts and pure compounds had varying effects on T cell ROS levels, migration, activation, and proliferation. Gene expression profiling using nCounter revealed differential regulation of several genes related to T cell functions, including activation and differentiation. Notably, apigenin reduced the GZMB induction in costimulated resting human T cells and the cytotoxic activity of activated human T cells.

One important aspect of this study was the use of primary human T cells instead of transformed cell lines. While several immortalized or tumor cell lines (such as Jurkat cells) are frequently used to investigate drug effects on T cells, due to their easy accessibility, and handling, they may exhibit altered signaling pathways compared to primary cells (45, 46). Thus, it appears important to utilize primary cells to obtain more representative and reliable results. Another crucial aspect of this study was the stimulation of the primary human T cells through CD3- and CD28- antibodies, which closely mimic *in vivo* activation by APC through the antigen receptor (TCR/CD3) and the costimulatory receptor CD28, respectively. In contrast, mitogens (such as PMA and Ionomycin, particularly in former times widely used in research to activate T cells) induce different gene profiles and functions in T cells (47).

Characterizing extracts is an essential component of studies that evaluate their bioactive potentials. Most characterized chamomile extracts in the literature were derived from aerial plant parts, especially the flower heads (9) and less from underground roots (48). The five chamomile extracts used in this study were derived from different parts of the plant and extracted by either water (CT and CR) or a water-ethanol mixture (CF, CRE, and CU). While the aqueous extracts were fermented, the hydro-ethanolic extracts were not fermented. The phytochemicals identified through LC/MS-MS analysis primarily comprised flavonoids, terpenoids, coumarins, caffeoylquinic acids and derivatives, as also reported in the literature for chamomile extracts (9, 26). Given the high heterogeneity of the different chamomile extracts and their differential effects on T cells described in this study, it is crucial to consider the potential interplay among the different phytochemicals in an additive, synergistic or antagonistic manner, and to take into account the extraction procedure including solvent controls.

T cells are constantly patrolling the entire body, searching for cognate antigen and inflamed sites (49). SDF-1α, part of the CXC chemokine subfamily, is a potent chemoattractant for peripheral blood lymphocytes (PBLs) including T and B cells via its corresponding chemokine receptor CXCR4 (44). Both SDF-1α and CXCR4 are constitutively expressed in various tissues (50). Although chamomile extracts were reported to have anti-chemotactic properties (51), our present study revealed that only the aq. root ferm extract (CR) inhibited the SDF-1α induced T cell migration. CR did not affect CXCR4 surface expression. This suggests that the CR extract suppresses downstream signaling processes in SDF-1α/CXCR4 induced T cell migration. One possibility would be an influence on SDF-1α induced Nitric oxide (NO) signaling (50). In this regard, a chamomile extract has been shown to inhibit nitric oxide signaling in murine macrophages (13). Furthermore, the disruption of actin cytoskeletal dynamics may provide an explanation for the inhibitory effect of CR extract on T cell migration. It may influence actin binding/bundling proteins such as L-plastin and cofilin, that are crucial for T cell activation and migration (52–54), and warrants further investigation.

Our study demonstrated a different inhibitory potential of chamomile extracts and pure compounds on T cell activation after CD3xCD28 costimulation. Three of the five chamomile extracts, namely aq. total ferm (CT), aq. root ferm (CR), and ethanolic flower (CF) had varying inhibitory effects on CD25 expression in costimulated T cells. However, ethanolic root (CRE) and ethanolic mother tincture (CU) had no effect on CD25 expression. The effects of the extracts on CD69 expression were less significant. Altogether, it implies that the bioactivities of chamomile extracts are influenced by the choice of extraction solvents and the stimulation method. Among the pure compounds, Ap and Cz exhibited more potent immunoinhibitory potentials on both CD25 and CD69 expressions as compared to the extracts, but the effect of the third pure compound Bis was rather inconsistent. Similar effects were observed on T cell proliferation capacities. The three extracts CT, CR, and CF significantly reduced the proliferation of CD3xCD28 costimulated T cells. In contrast, CU and CRE had no significant effect. Among the pure compounds, Ap and Cz significantly reduced T-cell proliferation, with a dose-dependent decrease observed only with Ap. The third pure compound Bis only showed a tendency to decrease T cell proliferation. The anti-proliferative effects of the potent extracts and pure compounds on human T cells can be attributed to the reduction of IL2 production, both intracellularly and extracellularly, as well as the decrease in surface expression of CD25, the high-affinity IL2 receptor. While previous studies have shown that chamomile essential oils can inhibit the inflammatory response of murine T cells (55), and the phytohemagglutinin-induced activation of the CD4^+^ subpopulation of human PBMCs (56), our study is the first to present a comprehensive evaluation of the effects of various extract types from different chamomile plant parts, as well as characteristic pure compounds of chamomile on human T cell activation and effector functions. The potent chamomile extracts and pure compounds lowered the production of the proinflammatory cytokine TNFα both intracellularly and extracellularly (by trend or significantly) but differentially affected IFNγ levels. It is important to note that trends with a lack of significance in the human system may be due to the high degree of donor variation, particularly in the extract treatment conditions. Surprisingly, both CT and CR significantly increased the proinflammatory cytokines IL6 and IL17F levels in the supernatants. This is in contrast to the anti-inflammatory effects of the extracts in general and specifically, their ability to reduce the levels of IL6 and IL17 in the serum of mice (55).

Chamomile extracts and pure compounds have been described as antioxidants, both *in vitro* using cell-free or cell-based assays, and *in vivo* (37–39, 57, 58). Their effects on ROS levels in T cells remained, however, unknown. While excessive and uncontrolled levels of ROS can have deleterious effects on cells through oxidative stress induction, low to moderate doses are required for intracellular signaling during both steady-state and antigen recognition processes (54, 59, 60). In the present study, we have determined the influence of the different chamomile extracts and pure compounds on the intracellular ROS levels in primary human T cells. Bis, in contrast to the other pure compounds, showed a slight increase in intracellular ROS in T cells. This prooxidative effect that had no significant effect on T cell functions differed from observations in neutrophils that indicated an antioxidant effect of Bis on the oxidative burst of neutrophils (37). The extracts CT, CR, and CF and the pure compounds Ap and Cz indeed exerted antioxidative effects in T cells. In a normal environment, insufficient ROS levels (specifically mitochondrial ROS) in the early phase of T cell stimulation can hamper the activation of T cells (61). This may provide one explanation for the inhibitory effects of the chamomile extracts and pure compounds on different T cell functions upon T cell costimulation. On the other hand, excessive ROS levels, as observed upon activation of neutrophils, or in a tumor microenvironment, can make T cells hyporesponsive (42, 43). It remains to be determined whether the antioxidative capacity of chamomile extracts and pure compounds may improve the activity of T cells in such a prooxidative microenvironment. Additionally, while ROS have been involved in mediating the suppressive function of Tregs (62), in aged Tregs inhibition of ROS could restore their suppressive activity (63). Thus, the antioxidative effects of the chamomile extracts and pure compounds might enhance the function of aged Tregs, while they may have an inhibitory effect on normal Tregs. Further investigations are required to elucidate the precise influence of the chamomile extracts and pure compounds on different Treg populations.

The nCounter gene expression profiling provided a broader molecular-level overview of the differential effects of the most potent chamomile extracts and pure compounds. Interestingly, *IL2* transcription was significantly downregulated by all the extracts and pure compounds. Thus, the inhibitory effects on T cell proliferation of these extracts and pure compounds may be explained by the transcriptional and translational downregulation of IL2 as well as the decreased CD25 (either by trend or significantly). Notably, there was a significant lack of overlap between the effects of the different extracts on the mRNA versus the protein levels of several other cytokines. There are several plausible explanations. The duration of costimulation is an important factor. The cells were subjected to 4 hours of costimulation before RNA isolation and gene expression analysis. However, for protein level detection, both intracellularly and extracellularly, a longer time point of 48 hours was used. Additionally, during the longer incubation period, the produced cytokines are also consumed by the cells for their own survival and proliferation. Also, mRNA levels do not necessarily reflect their corresponding protein levels when measured (64, 65). Variation between donors may be another factor that contributes to the differences in the transcriptional and translational data. Nevertheless, in line with the observed inhibitory effect on T cell activation, the IPA analysis based on the significantly regulated gene expression data revealed enrichment of pathways involving Th1/Th17 activation, IL17 signaling, and T cell differentiation as being downregulated by the potent chamomile extracts and pure compounds.

In the nCounter analysis of gene expression and the LEGENDplex™ cytokine measurement, among the most striking effects was the highly significant downregulation of GZMB by the pure compound Ap. These data were corroborated by the finding that Ap significantly reduced the induction of GZMB in CD3xCD28 costimulated T cells.

The cytotoxic activity of T cells is partially mediated by GZMB, which induces apoptosis in the target cells. Pre-formed GZMB are stored in lysosomal granules within the CTLs along with other cytotoxic molecules and are released during CTL degranulation in the synapse between the CTL and P815 tumor cells. In line with the finding that Ap decreased the GZMB induction in T cells, in addition, a decrease in the degranulation capacity of Ap treated CTLs was observed. Thereby, Ap treatment indeed reduced the killing capacity of CTLs towards P815 target cells. Interestingly, at a dose similar to that used in our study, Ap showed no significant effect on GZMB levels in human natural killer (NK) cells. In lower doses, Ap even increased GZMB levels in human NK cells (66). Moreover, similar doses of Ap showed no prominent effect neither on the degranulation nor the killing capacity of NK cells (66). Thus, Ap appears to diminish the cytotoxic activity of human T cells, but not of human NK cells.

While Ap has been widely known for its anti-inflammatory, anti-oxidative, anti-proliferative, and anti-cancer properties (67, 68), no study has yet demonstrated that apigenin can inhibit GZMB production in T cells and decrease the cytotoxic activity of CTLs. It is worth noting that CTLs can kill target cells through both granzyme-mediated and granzyme-independent pathways. The interaction between the Fas ligand (FasL) on the CTLs and the CD95 (Fas/Apo-1) receptor on the target cells induces apoptosis in the latter. However, since the target cells P815 lack CD95 expression (69, 70), the inhibitory effect of Ap on the CTL killing of P815 target cells was not due to influencing Fas-FasL mediated signaling pathways. In addition to its canonical function of cytotoxicity, GZMB also has non-canonical functions. It can escape into the extracellular environment of the tissues and cause tissue injury by influencing extracellular matrix remodeling and angiogenesis, which can cause chronic inflammation and eventually, an impaired tissue healing (71–73). Therefore, inhibiting GZMB production by T cells with Ap may have therapeutic effects for treating chronic inflammation in tissues.

## Conclusion

In conclusion, we have shown that chamomile derived extracts or pure compounds of chamomile inhibit the activation and cytotoxicity of primary human T cells. These findings offer novel molecular explanations for the clinically observed anti-inflammatory effects of chamomile and suggest a more extensive use of chamomile in T cell-driven chronic inflammatory diseases, such as chronic wounds and inflammatory skin diseases. Moreover, our data emphasize that for the evaluation of clinical studies, it is important to take into account the method of preparation of the extracts, as this results in a different phytochemical composition and thus, differential effects on T cell functions.

## Data availability statement

The data presented in the study are deposited in the MetaboLights repository, accession number MTBLS9800 and Gene Expression Omnibus (GEO) repository, accession GSE263440.

## Ethics statement

The studies involving humans were approved by Ethics Committee of Heidelberg University (S-089/2015). The studies were conducted in accordance with the local legislation and institutional requirements. The participants provided their written informed consent to participate in this study.

## Author contributions

DL: Writing – review & editing, Writing – original draft, Methodology, Investigation, Conceptualization. BW: Writing – review & editing, Methodology, Investigation. MS: Writing – review & editing, Methodology. BJ: Writing – review & editing, Methodology. HK: Writing – review & editing, Methodology. PW: Writing – review & editing, Methodology. KB: Writing – review & editing, Investigation. VB: Writing – review & editing, Funding acquisition. BN: Writing – review & editing, Methodology. EB: Writing – review & editing. YS: Writing – review & editing, Writing – original draft, Supervision, Resources, Funding acquisition, Conceptualization.
