# Supplementary material for: Comparative analysis of whole plant, flower and root extracts of Chamomilla recutita L. and characteristic pure compounds reveals differential anti-inflammatory effects on human T cells
Source: Front Immunol. 2024 Apr 24;15:1388962. doi: 10.3389/fimmu.2024.1388962 (PMC11077421; doi:10.3389/fimmu.2024.1388962)
Supplement: Supplementary file 1 [file DataSheet_1.pdf]

Supplementary Figure 1

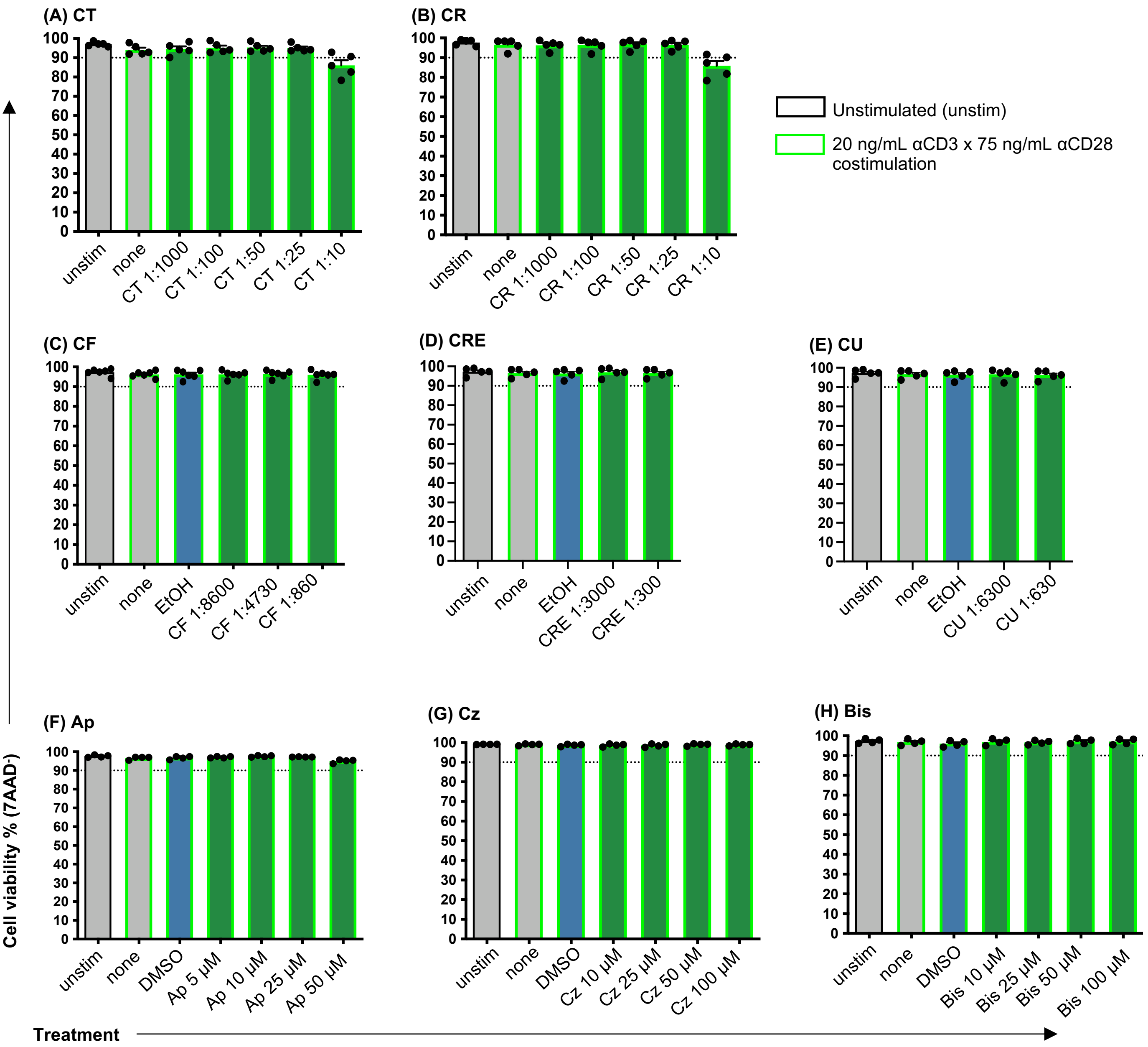

Suppl. Figure 1. Non-toxic doses of chamomile extracts are selected for further analysis (24 h). PBTs were kept untreated (none) or treated with solvent controls (EtOH/DMSO) or different dilutions/concentrations of chamomile extracts and pure compounds for 1 h. Next, they were assessed for their viability after 24 h co-stimulation, using 7-AAD staining. Extracts: (A) CT, (B) CR, (C) CF, (D) CRE, (E) CU. Pure compounds: (F) Ap, (G) Cz, (H) Bis. N= 4-6, each dot represents an individual donor. Black border = unstimulated (unstim). Green border = αCD3xCD28 costimulated T cells for 24 h. Statistical analysis was performed using One-way ANOVA, comparing against their respective controls (none/EtOH/DMSO) and the result expressed as Mean ± SEM.

Supplementary Figure 2

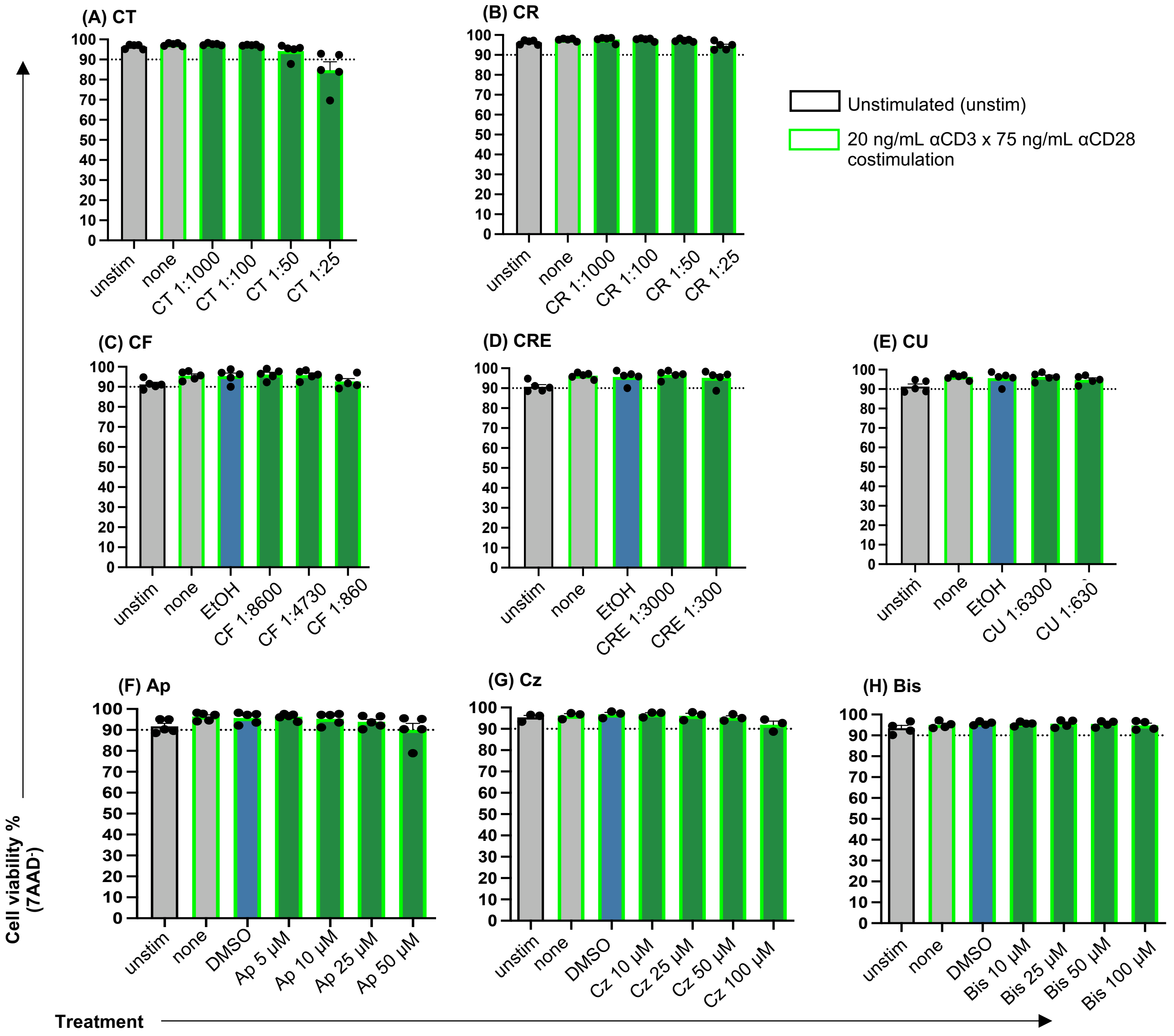

Suppl. Figure 2. Non-toxic doses of chamomile extracts are selected for further analysis (72 h). PBTs were kept untreated (none) or treated with solvent controls (EtOH/DMSO) or different dilutions/concentrations of chamomile extracts and pure compounds for 1 h. Next, they were assessed for their viability after 72 h co-stimulation, using 7-AAD staining. Extracts: (A) CT, (B) CR, (C) CF, (D) CRE, (E) CU. Pure compounds (F) Ap, (G) Cz, (H) Bis. N=3-5, each dot represents an individual donor. Black border = unstimulated (unstim). Green border = αCD3xCD28 costimulated T cells for 72 h. Statistical analysis was performed using One-way ANOVA, comparing against their respective controls (none/EtOH/DMSO) and the result expressed as Mean ± SEM.

Supplementary Figure 3

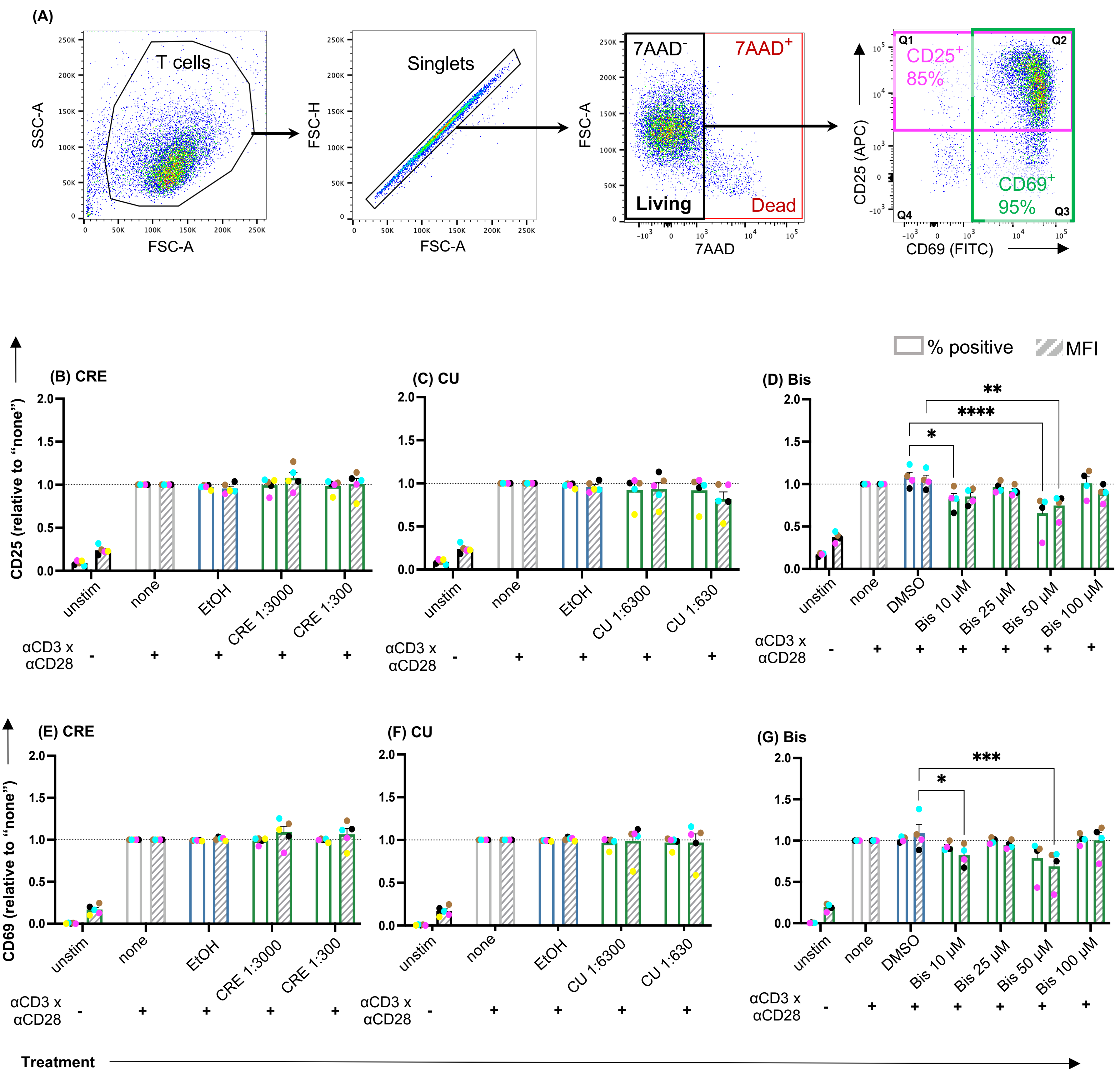

Suppl. Figure 3. The chamomile extracts CRE, CU and pure compound Bis show no prominent effects on CD25 and CD69 expressions. PBTs were kept untreated (none) or treated for 1 h and analysed for the surface expression of (B-D) CD25 and (E-G) CD69 after 24 h anti-CD3/CD28 co-stimulation. (A) Dotplots depicting the gating strategy for analyzing CD25 and CD69 expression, based on an untreated, co-stimulated sample. Extracts: (B,E) Ethanolic root (CRE), (C,F) ethanolic mother tincture (CU). Pure compound: (D,G) Bis. N = 4-5, each colored-dot represents an individual donor. (▭) = % positive. (▨) = geometric Mean Fluorescence Intensity (MFI). Data were normalized to the untreated samples (none). Statistical analysis was performed using Two-way ANOVA, comparing against their respective controls (none/EtOH/DMSO) and the result expressed as Mean ± SEM. * = p-value <0.05, ** = p-value <0.01, *** = p-value <0.001, ****=p-value <0.0001.

Supplementary Figure 4

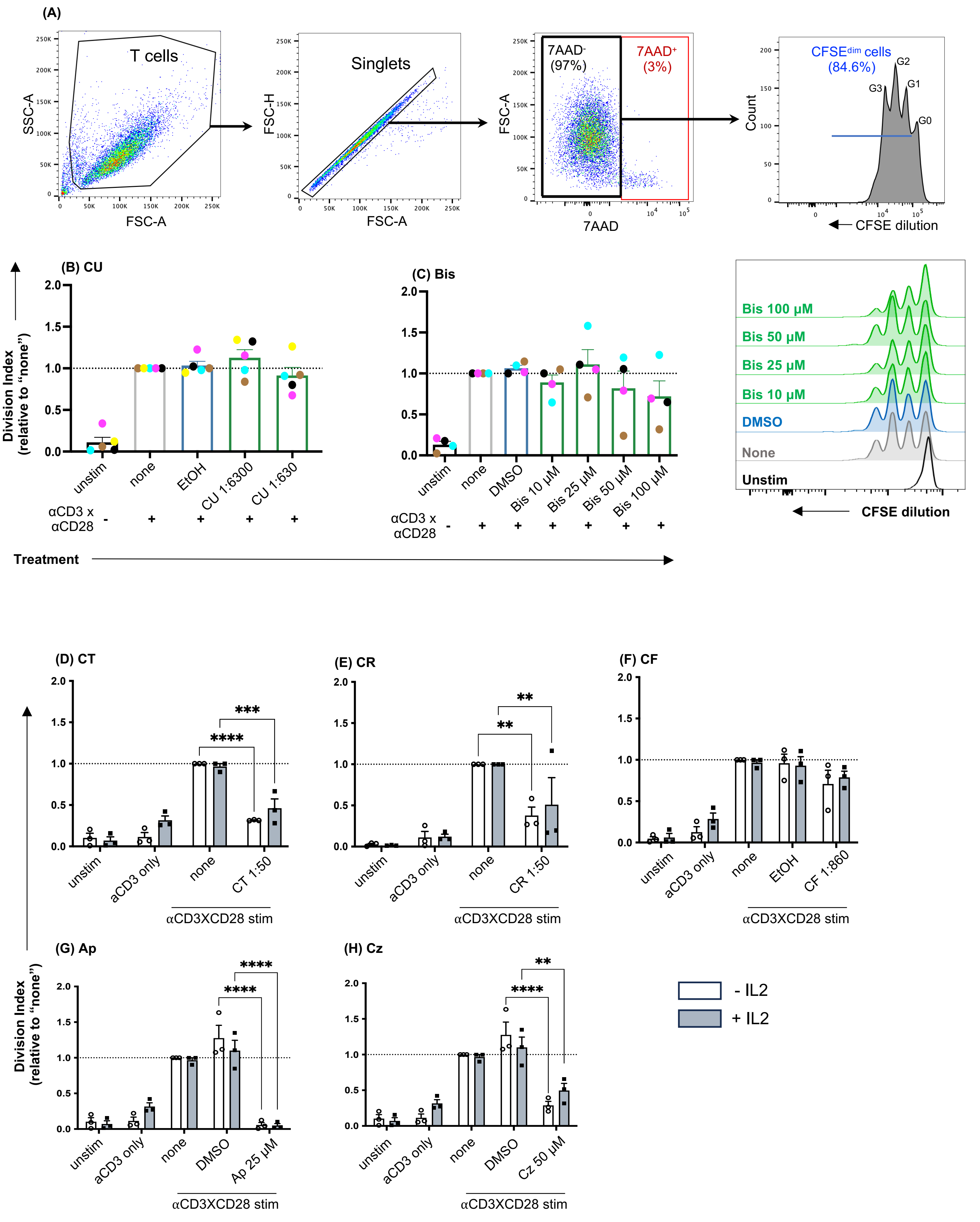

Suppl. Figure 4. The chamomile extract CU and pure compound Bis show no prominent effects on T cell proliferation, while exogenous IL2 supplementation does not rescue inhibition of T cell proliferation by CT, CR, CF, Ap and Cz. CFSE-stained PBTs were kept untreated (none) or treated with solvent controls (EtOH/DMSO) or different doses of extracts/ pure compounds for 1 h and analysed for (A-C) proliferation capacity and (D-H) rescue of proliferation by IL2 (40 U/mL) supplementation after 72 h anti-CD3/CD28 costimulation . (A) Dotplots and histogram depicting the gating strategy for analyzing T cell proliferation, based on an untreated, costimulated sample. G0 represents the undivided population, G1 represents the first generation, G2 represents the second generation and G3 represents the third generation. Extracts: (B) CU, (D), CT, (E) CR, (F) CF. Pure compounds: (C) Bis, (G) Ap, (H) Cz. N = 3-5, each dot represents an individual donor. Data were normalized to the untreated samples (none). Statistical analysis was performed using Two-way ANOVA, comparing against their respective controls (none/EtOH/DMSO) and the result expressed as Mean ± SEM. ** = p-value <0.01, *** = p-value <0.001, ****=p-value <0.0001.

Supplementary Figure 5

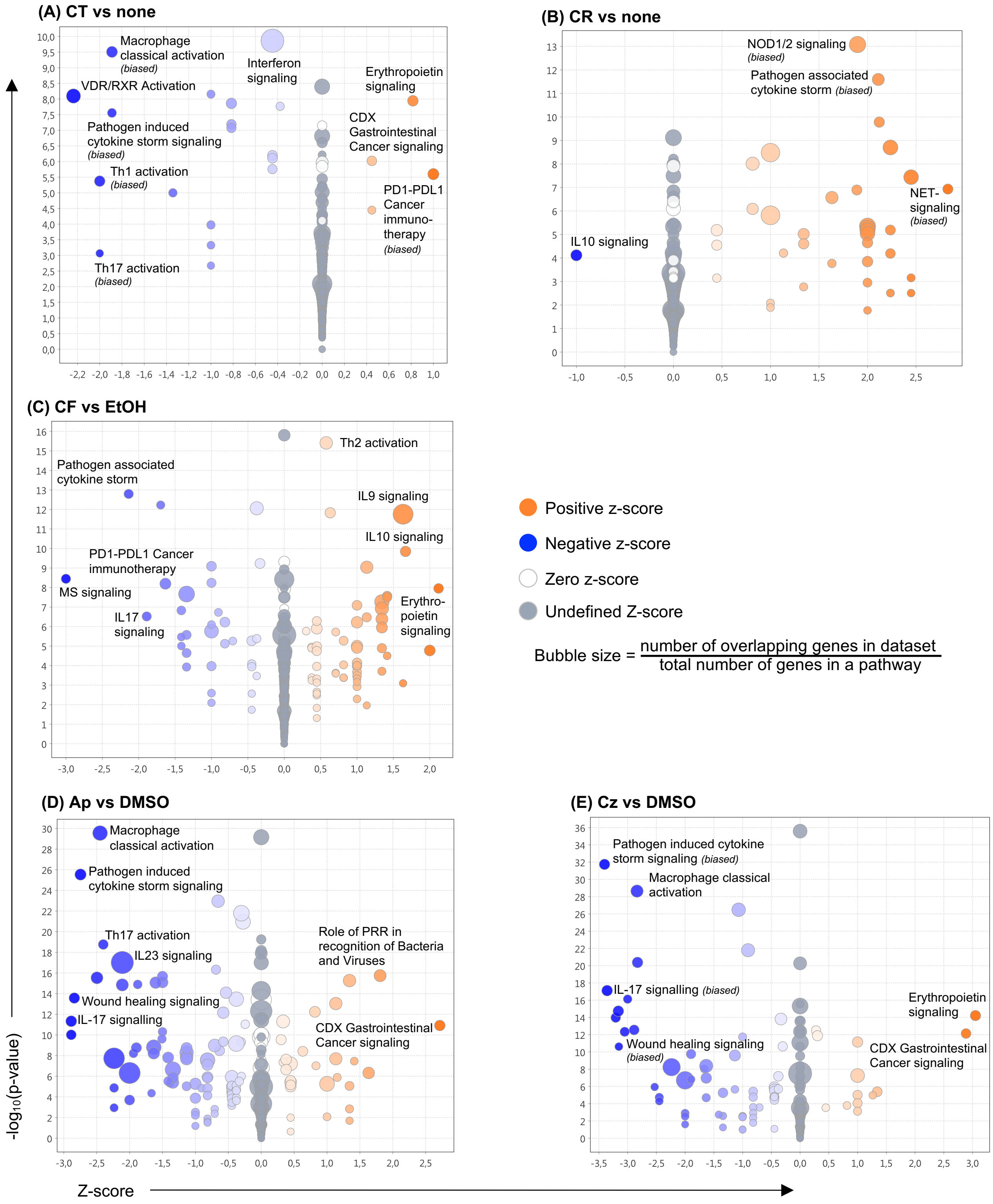

Suppl. Figure 5. Chamomile extracts and pure compounds are predicted to downregulate pathways associated with activation and differentiation by Ingenuity Pathway Analysis (IPA). The dataset comprising of gene identifiers and their corresponding log_2 expression fold ratios and p-values generated from nCounter analysis of chamomile extracts and lead compounds was uploaded into the web-based Ingenuity Pathway Analysis (IPA) software, and a core analysis was run with p-value cut-off value of 0.05. Under Canonical pathway module, the canonical pathways that were most enriched in each treated condition were displayed. A few top enriched pathways are shown for the extracts (A) CT, (B) CR, (C) CF and pure compounds (D) Ap (E) Cz. The p-values of overlap measures the overlapping of the genes in the dataset to those in a specific pathway. The activation z-scores measures the directional regulation of the dataset genes in a pathway and predicts if the pathway will be upregulated or downregulated. x-axis = z-score.(●) = z-score>0, (●) = z-score<0, (○) = z-score=0, (●) = z-score undefined). y-axis = -log10(p-value).

Supplementary Figure 6

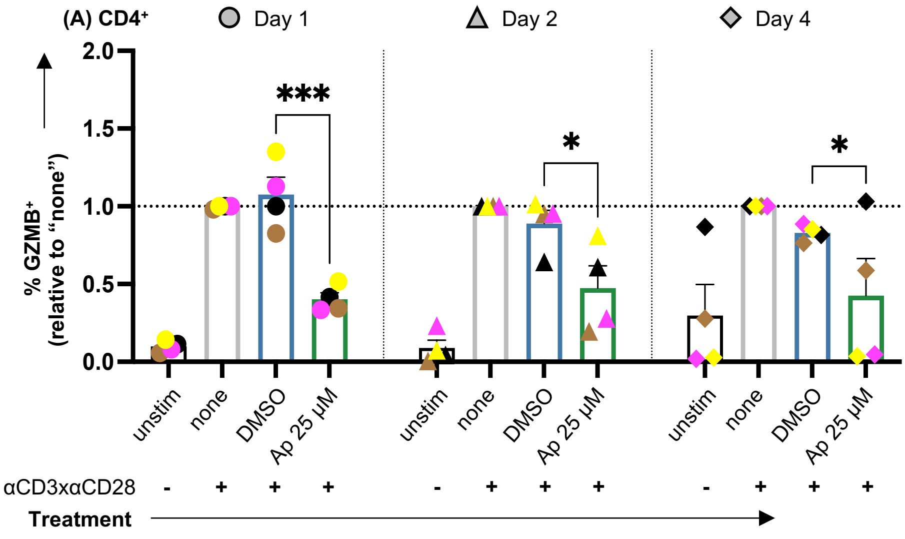

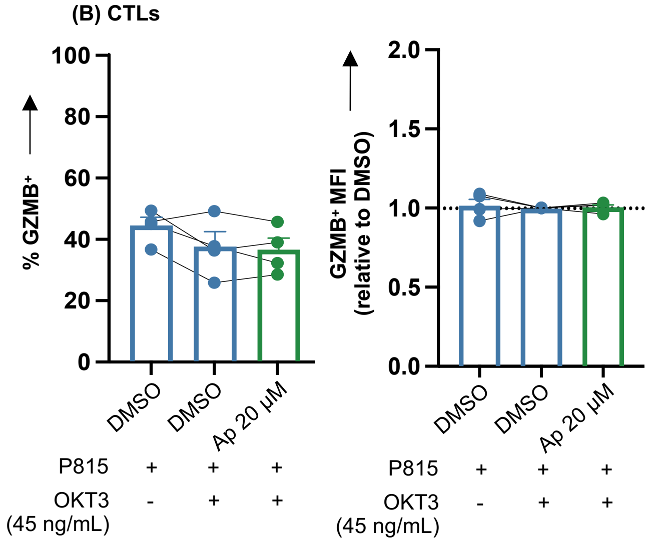

Suppl. Figure 6. Apigenin lowers granzyme B induction in CD4⁺ T cells but does not affect the level in CTLs. (A) Untreated or DMSO/apigenin-treated PBTs were co-stimulated with anti-CD3xCD28 for 1, 2 and 4 days. Monensin (3 µM) was added to the cell culture in the final 4 h costimulation. Afterwards, intracellular GZMB was detected in the costimulated T cells. (A) Ap treatment significantly decreased the intracellular GZMB level at all three time points in CD4⁺ T cells. (B) CTLs generated from PBTs (7 days co-stimulation) were kept untreated/ 1h treated with Ap and co-cultured with P815 cells (day 8). Ap treatment of CTLs had no effect on GZMB level (% and MFI). N=4. Each symbol represents an individual donor. Black bars = unstimulated (unstim), grey bars = untreated controls (none), blue bars = DMSO controls, green bars = Ap. Data for GZMB detection were normalized to respective controls. Statistical analysis using (B) One or (A) Two-way ANOVA was performed and results are expressed as Mean ± SEM. * = p-value < 0.05, *** = p-value < 0.001.
