# Supplementary material for: Comparative analysis of whole plant, flower and root extracts of Chamomilla recutita L. and characteristic pure compounds reveals differential anti-inflammatory effects on human T cells
Source: Front Immunol. 2024 Apr 24;15:1388962. doi: 10.3389/fimmu.2024.1388962 (PMC11077421; doi:10.3389/fimmu.2024.1388962)
Supplement: Supplementary file 2 [file Table_1.pdf]

SUPPLEMENTARY TABLES

Supplementary Table 1. LC-MS/MS results of the 5 examined commercial chamomile extracts with estimated quantities. CT: Chamomile aq. total ferm, CR: Chamomile aq. root ferm, CF: Chamomile ethanolic flower, CRE: Chamomile ethanolic root, CU: Chamomile mother tincture. * t_R is based on the MS data. Delay time 0.06 – 0.09 min; t_0 was not subtracted (1.85 min in PDA). **: Bold molecular ions belong to the dominant metabolite in the respective peak. 1: Minor metabolites with probably less importance, 2: Metabolites with lower concentrations, 3: Metabolites of importance, 4: Metabolites of probably high importance, X: Only optical absorption with average size; no ion peak in the TIC, XX: Only optical absorption with above-average size; no ion peak in the TIC.

Peak No.	Retention time, t_R [min]*	ESI mode	m/z		Tentative identification	Occurrence in *C. recutita* extracts				
			Precur-sor ion **	Fragmentation pattern MS2 (relative abundance [%])		CT	CR	CF	CRE	CU
**1.**	4.0	-	495.06	183.96 (4), 201.82 (8), 219.82 (33), 234.29 (2), 284.83 (4), 303.85 (5), 326.87 (1), 344.85 (100), 404.83 (63), 448.94 (2), 465.18 (1)	unknown	1				
**2.**	5.2	+	166.02	120.12 (100), 148.88 (9)	unknown	3	2	1		
**3.**	6.4	-	936.31	530.30 (100)	unknown	1	2			
**4.**	8.7	+/-	N/A	N/A	unknown		X			
**5.**	9.0	-	315.18	108.27 (7), 109.18 (15), 121.06 (4), 153.13 (100), 165.04 (12), 224.98 (8)	Protocatechuic acid *O*-hexoside (1)				1	
**6.**	9.2	-	153.10	109.22 (100)	Protocatechuic acid (2, 3)	1	1			
**7.**	10.3	-	218.91	111.07 (51), 172.13 (8), 172.88 (100)	unknown				1	
**8.**	12.2	+	146.24	71.83 (26), 75.01 (16), 83.83 (23), 97.53 (51), 127.70 (40), 129.06 (100)	unknown	1				
**9.**	12.2	-	353.14	135.22 (12), 179.10 (97), 191.22 (100)	Caffeoylquinic acid derivative (2, 4, 5)				2	
**10.**	13.8	+/-	N/A	N/A	unknown	X				
**11.**	14.9	+	217.03	132.22 (3), 144.23 (100), 159.85 (12), 171.13 (21), 173.03 (21), 188.03 (4)	unknown	1	1			
**12.**	16.2	+	231.03	158.17 (50), 187.11 (10), 188.06 (27), 214.02 (100)	unknown	1	2			
**13.**	17.9	+/-	N/A	N/A	unknown	X				
**14.**	18.6 – 18.7	-	353.10	173.26 (10), 179.25 (18), 191.22 (100)	Caffeoylquinic acid derivative (2, 4, 5)				4	2
			707.02	321.39 (4), 353.01 (100), 514.08 (12)	Caffeoylquinic acid derivative dimer					
**15.**	19.8	-	259.09	171.00 (3), 215.08 (100)	unknown	1				
**16.**	19.7	-	353.06	179.17 (10), 191.20 (100)	Caffeoylquinic acid derivative (2, 4, 5)			2		
			706.99	352.92 (100), 436.28 (14), 638.85 (19)	Caffeoylquinic acid derivative dimer					

Peak No.	Retention time, t_R [min]*	ESI mode	m/z		Tentative identification	Occurrence in *C. recutita* extracts				
			Precursor ion **	Fragmentation pattern MS2 (relative abundance [%])		CT	CR	CF	CRE	CU
**17.**	19.9	+	231.04	144.20 (4), 158.15 (57), 188.06 (36), 214.02 (100)	Unknown		1			
**18.**	20.0	-	221.14	206.09 (100)	unknown				2	
			428.92	206.10 (2), 221.12 (100), 382.69 (28)						
**19.**	20.8	-	355.08	149.13 (19), 192.99 (100)	Ferulic acid hexoside (2, 6)			1		
**20.**	21.6	-	595.23	355.18 (69), 385.21 (72), 415.24 (24), 457.15 (20), 475.08 (100), 487.18 (23), 505.07 (30), 577.16 (14)	Unknown (perhaps a flavonoid-di-gycoside)	1				
**21.**	21.7	-	355.05	193.0 149.13 (21), 192.99 (100)	Ferulic acid hexoside (2, 6)			3		3
			710.98	354.88 (100), 518.84 (10)	Ferulic acid hexoside dimer					
**22.**	24.8	-	165.09	119.23 (1), 147.01 (100)	unknown		2			
**23.**	27.7	-	593.28	353.20 (24), 383.22 (13), 473.17 (100), 503.21 (36), 575.17 (11)	Apigenin 6,8-di-*C*-hexoside (7, 8)	1				
**24.**	28.2 – 28.7	+	163.14	81.12 (4), 90.97 (34), 107.14 (14), 119.06 (100), 134.99 (9), 151.45 (8), 159.16 (30), 162.51 (5), 166.56 (4), 170.90 (53)	Perhaps umbelliferone (9)	2	2			
**25.**	28.3	-	559.21	160.05 (4), 175.04 (100), 193.07 (29), 337.14 (41), 383.08 (6), 499.19 (14), 517.14 (17)	unknown				2	
			604.86	272.11 (24), 343.75 (42), 354.59 (30), 370.82 (89), 431.47 (37), 452.36 (50), 458.92 (84), 507.34 (26), 514.13 (40), 532.85 (23), 537.03 (43), 559.20 (100), 587.12 (43), 587.61 (21)						
**26.**	28.7 – 29.1	-	639.18	315.20 (11), 431.15 (2), 477.18 (100), 519.12 (13), 561.17 (2)	unknown			1		2
**27.**	29.2	-	711.28	325.18 (1), 369.14 (7), 387.11 (3), 505.16 (2), 531.18 (26), 549.17 (100)	Ferulic acid hexoside dimer (2, 6)	2				
**28.**	29.7	-	355.04	149.14 (6), 193.02 (100)	Ferulic acid hexoside (2, 6)			2		
			711.04	324.94 (3), 354.98 (46), 369.16 (3), 387.02 (3), 418.71 (4), 531.12 (35), 549.20 (100)	Ferulic acid hexoside dimer (2, 6)					
**29.**	29.8	-	323.03	121.13 (4), 200.99 (100)	unknown	2				
			368.76	139.06 (19), 265.35 (22), 282.27 (28), 294.14 (13), 300.84 (24), 308.19 (39), 322.82 (100), 325.17 (36), 351.12 (39), 354.27 (74)						
**30.**	31.1	-	355.04	149.14 (7), 193.01 (100)	Ferulic acid hexoside (2, 6)			2		
			711.01	354.94 (100), 531.16 (4), 549.17 (10), 666.15 (2)	Ferulic acid hexoside dimer (2, 6)					
**31.**	30.9	-	367.08	111.09 (6), 154.87 (5), 173.04 (100), 193.04 (5)	unknown	1				
**32.**		-	355.06	149.14 (7), 193.01 (100)	Ferulic acid hexoside (2, 6)	2		2		

Peak No.	Retention time, t_R [min]*	ESI mode	m/z		Tentative identification	Occurrence in *C. recutita* extracts				
			Precursor ion **	Fragmentation pattern MS2 (relative abundance [%])		CT	CR	CF	CRE	CU
	32.1 – 32.3		711.08	354.35 (7), 354.96 (100), 368.99 (3), 531.13 (25), 549.25 (30), 693.41 (6)	Ferulic acid hexoside dimer (2, 6)					
**33.**	32.5 – 32.7	-	355.01	149.12 (6), 193.01 (100)	Ferulic acid hexoside (2, 6)			3	2	4
			710.97	354.91 (100), 549.23 (11)	Ferulic acid hexoside dimer (2, 6)					
**34.**	33.0	+	223.16	107.06 (7), 162.97 (45), 190.03 (19), 195.03 (25), 208.04 (100), 223.13 (64)	Perhaps isofraxidin (10)		2		2	
		-	221.12	206.07 (100), 207.09 (2)						
**35.**	34.3	-	711.29	271.17 (10), 325.10 (2), 369.00 (2), 387.11 (3), 433.05 (8), 531.12 (19), 549.20 (100), 693.18 (3)	unknown flavonoid di-glycoside derivative	2				
**36.**	34.6	+	194.98	177.04 (100)	unknown	1				
**37.**	34.8 – 35.0	-	233.11	127.12 (3), 143.12 (90), 157.20 (100), 186.87 (12)	unknown		2		2	
**38.**	34.8	-	711.26	271.08 (2), 369.06 (3), 387.08 (3), 531.14 (13), 549.18 (100)	unknown flavonoid di-glycoside derivative	2				1
**39.**	35.5	+	401.16	203.94 (41), 217.91 (17), 243.76 (37), 342.73 (15), 353.03 (51), 368.40 (12), 379.48 (12), 383.19 (28). 386.12 (78), 393.73 (100)	unknown		2			
**40.**	36.1 – 36.3	-	711.34	271.24 (15), 369.19 (31), 433.20 (7), 531.15 (100), 532.18 (5), 549.18 (60), 693.19 (4)	unknown flavonoid di-glycoside derivative	3				1
**41.**	36.4	-	195.11	151.15 (100), 177.06 (15)	unknown		3			
**42.**	36.7	-	233.17	148.83 (2), 162.17 (100)	unknown				2	
**43.**	36.7	-	711.34	271.19 (16), 369.14 (36), 433.11 (7), 531.13 (100), 549.16 (59)	unknown flavonoid di-glycoside derivative	3	2			
**44.**	36.9 - 37.1	-	711.27	271.13 (12), 369.10 (33), 531.11 (100), 549.18 (58)	unknown flavonoid di-glycoside derivative		1			1
**45.**	37.2	+	401.18	203.98 (42), 244.26 (48), 311.29 (15), 353.02 (30), 385.96 (100), 387.06 (31), 393.33 (22)	unknown		2			
**46.**	37.6	-	493.21	316.17 (2), 331.15 (100), 373.09 (8)	Petuletin glucoside (7)			2		
**47.**	37.7	-	711.34	271.16 (24), 325.19 (4), 387.24 (2), 433.06 (20), 531.11 (39), 549.22 (100), 693.10 (13)	unknown flavonoid di-glycoside derivative	2				
**48.**	37.9	-	559.28	175.09 (33), 193.06 (36), 265.03 (10), 337.11 (48), 499.18 (100), 517.22 (85)	unknown				2	
			604.92	323.31 (36), 330.10 (34), 347.50 (100), 352.14 (26), 440.11 (37), 443.87 (86), 530.05 (30), 535.71 (30), 541.26 (30), 545.37 (20), 559.28 (74), 586.86 (66)						
**49.**	38.1	-	447.23	285.25 (100)	Luteolin glycoside (8)					2

Peak No.	Retention time, t_R [min]*	ESI mode	m/z		Tentative identification	Occurrence in *C. recutita* extracts				
			Precursor ion **	Fragmentation pattern MS2 (relative abundance [%])		CT	CR	CF	CRE	CU
			493.03	315.10 (2), 329.52 (4), 331.11 (100), 373.17 (4)	Petuletin glucoside (8)					
			**593.25**	285.23 (100), 447.14 (2)	Luteolin di-glycoside (8)					
**50.**	38.3	-	549.25	193.05 (9), 256.19 (17), 271.11 (100), 310.22 (4), 325.13 (6), 343.13 (18), 369.02 (75), 387.12 (81), 531.12 (7)	unknown flavonoid glycoside derivative	2				
**51.**	38.6	-	261.21	125.17 (19), 169.01 (4), 187.15 (100)	unknown				2	
**52.**	38.9	-	**447.25**	285.24 (100), 327.13 (3)	Luteolin glycoside (8)					2
			493.02	316.17 (2), 331.13 (100), 373.05 (5)	Petuletin glucoside (7)					
			593.23	217.21 (2), 285.19 (100), 393.36 (2), 446.35 (4), 504.21 (2)	Luteolin di-glucoside (8)					
**53.**	39.1	-	447.31	285.25 (100), 327.16 (3)	Luteolin glucoside (8)			3		
			**493.23**	316.21 (2), 331.16 (100), 373.07 (7)	Petuletin glucoside (7)					
			711.34	271.13 (14), 325.08 (3), 343.17 (3), 369.11 (18), 433.04 (15), 531.13 (58), 549.21 (100), 693.13 (9)	unknown flavonoid di-glycoside derivative					
**54.**	39.4 – 40.0	-	549.27	193.04 (8), 256.18 (17), 271.09 (100), 310.17 (4), 325.07 (6), 343.13 (22), 369.02 (71), 387.10 (100), 531.06 (4)	unknown flavonoid glycoside derivative	2	2			
**55.**	39.8 – 40.2	-	711.32	271.14 (13), 369.08 (14), 433.10 (15), 531.12 (45), 549.20 (100), 693.16 (5)	unknown flavonoid di-glycoside derivative	2		2		
**56.**	40.3	-	711.34	271.21 (7), 325.23 (5), 369.01 (16), 432.93 (23), 531.10 (52), 549.17 (100), 693.10 (7)	unknown flavonoid di-glycoside derivative		2			1
**57.**	40.4	-	187.23	125.15 (100), 142.69 (3), 168.92 (8), 187.22 (3)	Azelaic acid (11)				2	
			567.03	270.00 (3), 329.28 (4), 348.07 (3), 393.07 (3), 519.99 (5), 521.08 (100)	unknown					
**58.**	41.5 – 41.6	-	515.15	179.18 (8), 299.01 (4), 335.03 (4), 353.09 (100)	Dicaffeoylquinic acid derivative (5)			2		2
			711.28	271.10 (27), 369.06 (42), 432.99 (10), 505.03 (7), 531.09 (100), 549.17 (81)	unknown flavonoid di-glycoside derivative					
**59.**	41.3	+	174.85	138.98 (3), 156.90 (100), 165.18 (2)	unknown	2				
**60.**	41.4	-	515.25	173.32 (6), 179.26 (8), 203.28 (7), 255.36 (3), 299.25 (6), 317.16 (3), 335.35 (9), 353.10 (100)	Dicaffeoylquinic acid derivative (5)				3	
**61.**	42.3 – 42.7	-	711.32	271.40 (29), 325.63 (4), 343.36 (4), 369.36 (45), 433.33 (15), 505.41 (3), 531.27 (100), 549.22 (100)	unknown flavonoid di-glycoside derivative	4				3
			431.30	269.27 (100), 311.14 (8)	Apigenin glucoside derivative (12)			3		
**62.**	42.7 – 43.0	-	515.13	191.32 (1), 335.38 (2), 353.10 (100)	Dicaffeoylquinic acid derivative (5)				3	

Peak No.	Retention time, t_R [min]*	ESI mode	m/z		Tentative identification	Occurrence in *C. recutita* extracts				
			Precursor ion **	Fragmentation pattern MS2 (relative abundance [%])		CT	CR	CF	CRE	CU
**63.**	43.0	-	711.33	271.18 (31), 325.14 (6), 343.19 (5), 369.12 (47), 433.10 (15), 505.20 (4), 531.12 (100), 549.19 (88)	unknown flavonoid di-glycoside derivative		4			
**64.**	43.3	-	193.06	149.11 (100)	Ferulic acid	1				
**65.**	44.1	-	**431.27**	268.39 (11), 269.26 (100), 270.24 (5), 311.13 (8)	Apigenin glucoside derivative (12)					3
			447.11	300.16 (4), 315.15 (100), 357.16 (10)	Methyl ellagic acid pentoside (12, 13)					
**66.**	44.6 – 45.0	-	**431.32**	269.25 (100), 311.15 (7)	Apigenin glucoside derivative (12)			3		2
			**447.09** (only CU)	285.23 (100)	Luteolin-*O*-hexose (2, 14)					
			461.22 (only CU)	299.18 (100), 313.52 (3), 371.12 (3), 415.02 (3), 446.17 (49), 447.16 (5)	Chrysoeriol-7-*O*-glycoside (15)					
			**477.19**	314.17 (19), 315.17 (100), 357.05 (6)	Isorhamnetin glucoside (7)					
**67.**	45.5	-	Like 66.	Like 66.	Like 66.			2		
**68.**	45.9	-	711.34	549.2 387.19 (6), 531.22 (11), 549.20 (100), 693.14 (2)	unknown flavonoid di-glycoside derivative	3				
**69.**	46.0 – 46.4	-	461.26	284.25 (3), 299.17 (100), 341.05 (3), 446.15 (56)	Chrysoeriol-7-*O*-glycoside (15)			1		2
**70.**	46.3 – 47.0	-	515.19	179.27 (3), 203.30 (17), 255.37 (7), 299.26 (16), 317.25 (8), 335.34 (3), 353.11 (100)	Dicaffeoylquinic acid derivative (5)			2	3	2
**71.**	46.7	-	711.25	387.23 (4), 531.14 (8), 549.19 (100), 692.99 (2)	unknown flavonoid di-glycoside derivative		1			
**72.**	48.1	-	515.18	179.08 (2), 203.16 (14), 255.20 (6), 299.05 (14), 317.00 (6), 335.14 (3), 353.10 (100)	Dicaffeoylquinic acid derivative (5)				2	
**73.**	48.4	-	667.21	343.14 (5), 505.15 (100)	unknown	1				
**74.**	49.6 – 50.1	-	639.36	315.23 (3), 493.16 (8), 519.33 (100)	unknown	XX	XX	2		1
**75.**	51.3	-	655.24	208.98 (4), 270.22 (1), 287.20 (1), 316.17 (16), 323.16 (13), 331.15 (100), 492.96 (1), 505.17 (3), 533.13 (20)	Pentahydroxymethoxyflavone caffeoylglucoside (7)			2		
**76.**	51.5	-	693.39	337.07 (4), 499.23 (57), 513.12 (8), 517.22 (100)	unknown				2	
			738.3	337.05 (53), 403.32 (45), 417.50 (30), 512.95 (25), 546.28 (56), 559.36 (47), 561.45 (20), 577.14 (98), 649.26 (100), 675.73 (55), 677.30 (49), 694.04 (27), 720.73 (43)						
**77.**	52.0	+	455.24	437.2 351.21 (5), 367.31 (2), 368.25 (9), 369.22 (16), 419.19 (3), 437.20 (100)	unknown	2				
**78.**	52.1	-	473.32	269.3 269.29 (100), 311.17 (3), 413.22 (31)	Apigenin acetyl-glucoside derivative (12)			1		

Peak No.	Retention time, t_R [min]*	ESI mode	m/z		Tentative identification	Occurrence in *C. recutita* extracts				
			Precursor ion **	Fragmentation pattern MS2 (relative abundance [%])		CT	CR	CF	CRE	CU
**79.**	52.3	+	455.21	351.23 (4), 369.19 (17), 419.22 (2), 437.20 (100)	unknown		2			
**80.**	53.8	-	473.33	269.29 (100), 311.20 (3), 323.22 (1), 413.24 (27)	Apigenin acetyl-glucoside derivative (12)			2		
**81.**	53.5	-	413.13	345.25 (8), 367.14 (100)	unknown	2				
**82.**	53.7 – 53.8	-	207.23	135.35 (4), 161.29 (8), 179.21 (48), 207.19 (100)	unknown				2	2
**83.**	54.5	-	739.20	531.1 271.15 (4), 369.10 (7), 433.09 (3), 531.06 (100), 577.14 (57), 693.01 (17)	unknown flavonoid di-glycoside derivative					3
**84.**	54.8	+	401.05	167.06 (6), 247.05 (4), 330.14 (11), 351.06 (11), 369.03 (27), 383.07 (100)	unknown				1	
**85.**	55.3	-	739.20	531.1 369.07 (5), 433.06 (3), 531.07 (100), 577.18 (47), 693.03 (17)	unknown flavonoid di-glycoside derivative			1		
**86.**	56.7	-	409.09	118.88 (6), 130.82 (5), 143.01 (6), 160.91 (32), 178.82 (100), 200.99 (72), 363.02 (14)	unknown				2	
			735.28	480.96 (3), 499.23 (9), 513.25 (8), 517.23 (7), 527.16 (3), 541.20 (24), 559.19 (100), 675.24 (56), 693.25 (54)						
**87.**	59.5	-	735.30	559.2 337.26 (3), 499.34 (5), 513.30 (9), 527.36 (3), 541.31 (53), 559.21 (100), 675.20 (11), 693.21 (11)	unknown				3	
**88.**	59.7 – 60.0	-	473.33	269.37 (100), 311.25 (11), 341.27 (3), 413.30 (6)	Apigenin acetyl-glucoside derivative (12)			3		2
**89.**	60.3	-	473.34	269.30 (100), 311.16 (4), 413.24 (20)	Apigenin acetyl-glucoside derivative (12)			2		
**90.**	60.7	-	473.34	269.31 (100), 311.18 (3), 413.23 (30)	Apigenin acetyl-glucoside derivative (12)			2		
**91.**	61.9	-	861.30	271.15 (10), 325.15 (5), 369.07 (8), 421.07 (10),475.23 (5), 519.18 (65), 531.17 (3), 549.22 (6), 583.00 (4), 681.17 (55), 682.23 (7), 699.26 (100)	unknown	2				
**92.**	61.9	-	469.21	188.95 (3), 219.12 (100)	unknown				2	
			735.34	499.18 (12), 517.17 (17), 527.15 (4), 541.19 (55), 559.17 (100), 675.27 (4), 693.19 (33)						
**93.**	62.8	+	607.24	365.42 (5), 410.09 (10), 446.38 (8), 450.25 (100), 463.31 (6)	unknown		1			
**94.**	64.6 – 64.7	-	555.04	202.61 (6), 467.97 (7), 473.43 (5), 491.29 (19), 509.10 (100), 554.59 (5)	unknown		X		1	
**95.**	65.4	-	417.19	195.07 (4), 355.21 (3), 399.16 (100)	unknown		1			

Peak No.	Retention time, t_R [min]*	ESI mode	m/z		Tentative identification	Occurrence in *C. recutita* extracts				
			Precur-sor ion **	Fragmentation pattern MS2 (relative abundance [%])		CT	CR	CF	CRE	CU
**96.**	65.2 – 65.7	-	327.33	171.33 (44), 209.55 (6), 211.37 (33), 221.34 (33), 229.31 (100), 239.29 (40), 273.34 (6), 291.28 (83), 307.19 (6), 309.25 (44)	unknown	3	1			
**97.**	65.3 – 65.5	-	269.27	149.07 (1), 225.25 (2), 269.21 (100)	Apigenin (standard)			2		1
**98.**	66.1	-	327.30	155.21 (7), 171.17 (46), 201.24 (6), 209.29 (10), 211.20 (20), 221.14 (32), 227.32 (5), 229.27 (82), 239.24 (38), 273.27 (6), 291.26 (100), 307.20 (6), 309.22 (69), 327.25 (69)	unknown	2				
**99.**	67.0	-	299.18	284.18 (100)	Hispidulin (12)					2
**100.**	67.4	+/-	N/A	N/A	unknown	X				
**101.**	67.6	+	201.08	66.98 (13), 91.17 (7), 105.12 (12), 117.14 (23), 131.14 (32), 145.10 (61), 159.02 (100), 173.06 (77). 186.12 (7)	unknown	2				
**102.**	67.7	-	785.39	545.35 (16), 639.32 (11), 665.28 (100)	Tetra-*cis*/*trans*-coumaroyl polyamine derivative (15-17)					2
**103.**	67.8	-	777.29	541.63 (9), 559.59 (17), 583.53 (36), 601.30 (100), 717.29 (8), 735.26 (39)	unknown				3	
**104.**	67.9	-	315.26	300.21 (100), 315.16 (82)	Isorhamnetin (9, 18)			1		
			**785.47**	545.34 (15), 639.41 (11), 665.32 (100)	Tetra-*cis*/*trans*-coumaroyl polyamine derivative (15-17)					
**105.**	68.8	-	785.36	545.32 (15), 639.39 (11), 665.30 (100)	Tetra-cis/trans-coumaroyl polyamine derivative (15-17)					1
**106.**	69.0 – 69.2	-	329.34	171.60 (15), 211.21 (8), 211.63 (45), 229.37 (100), 293.37 (15), 309.37 (10), 311.35 (40)	Cirsiliol derivative (19, 20)	3	3	2	2	1
**107.**	69.3 – 69.5	-	329.32	171.22 (24), 211.22 (18), 229.26 (55), 293.30 (20), 309.23 (8), 310.19 (6), 311.29 (35)	Cirsiliol derivative (19, 20)	2	1			
**108.**	69.7 – 70.0	-	785.8	545.33 (15), 639.36 (9), 665.29 (100)	Tetra-*cis*/*trans*-coumaroyl polyamine derivative (15-17)			1		2
**109.**	69.4 – 69.9	-	329.26	127.35 (3), 171.35 (13), 185.32 (2), 193.30 (2), 200.82 (1), 201.91 (4), 211.31 (11), 212.02 (2), 229.15 (56), 283.01 (6), 283.51 (1), 293.28 (12), 309.09 (8), 309.83 (3), 311.26 (51)	Cirsiliol derivative (19, 20)	1	1		1	
**110.**	70.2 – 70.6	-	417.09	309.17 (4), 343.21 (2), 355.19 (37), 373.06 (100), 387.06 (2)	unknown	1	1			

Peak No.	Retention time, t_R [min]*	ESI mode	m/z		Tentative identification	Occurrence in *C. recutita* extracts				
			Precursor ion **	Fragmentation pattern MS2 (relative abundance [%])		CT	CR	CF	CRE	CU
**111.**	70.2	-	785.46	545.39 (13), 639.39 (11), 665.32 (100)	Tetra-*cis*/*trans*-coumaroyl polyamine derivative (15-17)			1		
**112.**	70.3 – 70.7	-	785.42	545.26 (10), 639.41 (10), 665.28 (100)	Tetra-*cis*/*trans*-coumaroyl polyamine derivative (15-17)			3		2
**113.**	71.2 – 71.3	-	785.45	545.71 (15), 639.79 (11), 665.42 (100)	Tetra-*cis*/*trans*-coumaroyl polyamine derivative (15-17)			4		3
**114.**	71.9	+	465.32	379.23 (15), 407.24 (8), 419.19 (30), 447.25 (100)	unknown					1
**115.**	73.4	+/-	N/A	N/A	unknown	X				
**116.**	75.4	-	233.02	189.1107.06 (5), 143.34 (4), 147.13 (7), 163.20 (5), 171.09 (77), 189.12 (100)	unknown	2				
**117.**	76.6 – 76.9	-	373.17	358.11 (100)	unknown			1		1
**118.**	77.0	+	358.20	315.11 (5), 328.08 (4), 343.07 (100)	unknown					2
			429.12	181.06 (16), 219.05 (5), 233.01 (6), 261.05 (5), 358.10 (33), 380.20 (11), 401.01 (6), 411.07 (100)						
**119.**	77.4 – 77.6	-	293.08	193.06 (1), 221.36 (18), 236.13 (100)	unknown	2	1			
**120.**	78.9	+	316.26	280.19 (60), 298.13 (100)	unknown			1		3
**121.**	79.3	-	823.66	521.61 (43), 589.46 (33),615.47 (29), 647.43 (53), 673.30 (26), 691.36 (39), 699.52 (50), 735.51 (26), 743.31 (27), 751.47 (47), 761.44 (49), 769.46 (91), 787.35 (100), 805.51 (62)	unknown			1		
**122.**	79.6 – 79.7	-	461.24	170.39 (15), 181.03 (37), 279.38 (100), 284.95 (22), 299.03 (21), 409.92 (36), 417.30 (42)	unknown			2		1
			779.61	521.50 (39), 547.27 (20), 563.49 (24), 573.62 (28), 579.38 (23), 589.48 (52), 641.60 (20), 648.43 (26), 673.55 (56), 717.49 (25), 725.42 (100), 735.63 (38), 743.49 (74), 761.45 (30)						
			**823.68**	521.43 (16), 563.49 (28), 607.42 (14), 615.56 (19), 647.48 (41), 673.40 (27), 699.40 (33), 717.20 (15), 751.45 (62), 761.52 (20), 769.46 (67), 779.49 (26), 787.46 (100), 805.47 (84)						
**123.**	80.5	-	461.25	279.26 (75), 418.21 (43), 445.17 (100)	unknown					1
**124.**	84.5 – 84.7	-	461.31	153.00 (38), 181.03 (58), 279.33 (100), 279.57 (52), 402.03 (85), 403.04 (17), 415.17 (49), 432.09 (13), 433.24 (60), 442.98 (50), 461.29 (61)	unknown			1	X	3

Peak No.	Retention time, t_R [min]*	ESI mode	m/z		Tentative identification	Occurrence in *C. recutita* extracts				
			Precursor ion **	Fragmentation pattern MS2 (relative abundance [%])		CT	CR	CF	CRE	CU
			557.26	423.39 (1), 453.33 (100), 454.34 (8), 471.76 (1), 497.17 (31), 498.26 (6), 515.27 (19), 516.30 (4)						
			721.17	512.88 (3), 615.24 (6), 675.10 (100)						3
**125.**	86.5	+	221.00	109.06 (4), 119.13 (29), 126.89 (34), 132.99 (11), 146.95 (8), 149.09 (5), 160.97 (4), 203.00 (100)	unknown	2		2		1
**126.**	87.8 – 88.1	-	461.30	181.12 (44), 198.87 (38), 279.23 (100), 433.60 (34), 446.03 (58)	unknown			1		
			557.15	453.29 (42), 497.12 (100), 513.72 (2)						
			723.21	529.03 (7), 541.34 (12), 677.08 (100), 691.93 (9)						2
**127.**	88.8 – 89.0	-	559.18	253.15 (2), 513.05 (100)	unknown			1		2
**128.**	89.5	+	221.06	119.13 (28), 126.89 (32), 132.97 (9), 146.96 (8), 149.06 (6), 203.01 (100)	unknown			2		2
**129.**	89.9 – 90.0	-	559.25	513.15 (100)	unknown			2		3
**130.**	92.8	-	977.36	577.27 (7), 697.23 (26), 857.23 (100), 875.31 (13)	unknown				1	
**131.**	93.8 – 94.1	-	577.40	225.11 (13), 299.19 (14), 577.39 (100), 578.38 (44)	unknown			1		2
**132.**	94.0	-	561.25	515.08 (100)	unknown					2
**133.**	95.0	+	295.20	179.06 (7), 276.95 (100)	unknown				2	
**134.**	95.9	+	295.18	277.04 (100)	unknown				2	
**135.**	106.1	+	405.39	183.12 (10), 249.17 (24), 263.05 (14), 267.06 (65), 281.07 (35), 309.12 (12), 319.19 (19), 323.17 (18), 377.22 (29), 387.20 (100)	unknown			1		3
**136.**	106.7	-	271.34	225.38 (6)	unknown			2		2
**137.**	111.1	+	609.58	515.43 (6), 531.33 (32), 559.27 (14), 577.28 (8), 591.24 (100)	unknown			1		
**138.**	111.2	+	639.58	565.39 (100), 579.33 (5), 593.18 (5)	unknown					2
			653.51	579.37 (100), 580.38 (20), 593.34 (7),635.27 (5)						
**139.**	111.9	-	459.31	180.95 (21), 199.07 (10), 277.25 (100), 413.12 (6)	unknown			2		2
**140.**	112.9	+	282.37	247.17 (77), 265.07 (100)	unknown			2		
**141.**	113.4	+	609.59	515.38 (2), 531.34 (21), 559.28 (6), 591.24 (100)	unknown			2		
**142.**	115.8	+	593.62	533.37 (92)	unknown			2		
**143.**	117.2	-	680.76	383.44 (60), 426.40 (9), 438.37 (7)	unknown			1		
			726.40	527.35 (68), 537.00 (56), 555.16 (37), 590.51 (18), 596.36 (43), 600.26 (35), 679.71 (100), 698.23 (18)						

References

1. Shrestha A. Phytochemical Analysis of Rhododendron Species. [PhD Thesis in Chemistry]. [Bremen]: Jacobs University; 2016.
2. Guimaraes R, Barros L, Duenas M, Calhelha RC, Carvalho AM, Santos-Buelga C, et al. Infusion and decoction of wild German chamomile: Bioactivity and characterization of organic acids and phenolic compounds. Food Chem. 2013;136(2):947-54.
3. Caleja C, Barros L, Antonio AL, Ciric A, Barreira JCM, Sokovic M, et al. Development of a functional dairy food: Exploring bioactive and preservation effects of chamomile (Matricaria recutita L.). J Funct Foods. 2015;16:114-24.
4. Willems JL, Khamis MM, Saeid WM, Purves RW, Katselis G, Low NH, et al. Analysis of a series of chlorogenic acid isomers using differential ion mobility and tandem mass spectrometry. Anal Chim Acta. 2016;933:164-74.
5. Sharopov F, Wetterauer B, Gulmurodov I, Khalifaev D, Safarzoda R, Sobeh M, et al. Chlorogenic and 1,5-Dicaffeoylquinic Acid-Rich Extract of Topinambur (Helianthus tuberosus L.) Exhibits Strong Antioxidant Activity and Weak Cytotoxicity. Pharm Chem J+. 2020;54(7):745-54.
6. Weber B, Herrmann M, Hartmann B, Joppe H, Schmidt CO, Bertram HJ. HPLC/MS and HPLC/NMR as hyphenated techniques for accelerated characterization of the main constituents in Chamomile (Chamomilla recutita [L.] Rauschert). Eur Food Res Technol. 2008;226(4):755-60.
7. Lin LZ, Harnly JM. LC-PDA-ESI/MS Identification of the Phenolic Components of Three Compositae Spices: Chamomile, Tarragon, and Mexican Arnica. Nat Prod Commun. 2012;7(6):749-52.
8. Geng P, Sun JH, Zhang ML, Li XN, Harnly JM, Chen P. Comprehensive characterization of C-glycosyl flavones in wheat (Triticum aestivum L.) germ using UPLC-PDA-ESI/HRMSn and mass defect filtering. J Mass Spectrom. 2016;51(10):914-30.
9. Novakova L, Vildova A, Mateus JP, Goncalves T, Solich P. Development and application of UHPLC-MS/MS method for the determination of phenolic compounds in Chamomile flowers and Chamomile tea extracts. Talanta. 2010;82(4):1271-80.
10. Majnooni MB, Fakhri S, Shokoohinia Y, Mojarrab M, Kazemi-Afrakoti S, Farzaei MH. Isofraxidin: Synthesis, Biosynthesis, Isolation, Pharmacokinetic and Pharmacological Properties. Molecules. 2020;25(9).
11. Abdelaziz S, Elhassanny WHBHAEM, Al-Yousef HM, Elsayed MA, Adel R. Ultra performance liquid chromatography-tandem mass spectrometeric analysis of ethyl acetate fraction from saudi Lavandula coronopifolia Poir and evaluation of its cytotoxic and antioxidant activities. Journal of Herbmed Pharmacology. 2020;9(3):268-76.
12. Martins N, Barros L, Santos-Buelga C, Henriques M, Silva S, Ferreira ICFR. Evaluation of bioactive properties and phenolic compounds in different extracts prepared from Salvia officinalis L. Food Chem. 2015;170:378-85.
13. Chiste RC, Mercadante AZ. Identification and quantification, by HPLC-DAD-MS/MS, of carotenoids and phenolic compounds from the Amazonian fruit Caryocar villosum. J Agric Food Chem. 2012;60(23):5884-92.
14. Tahir NI, Shaari K, Abas F, Parveez GK, Ishak Z, Ramli US. Characterization of apigenin and luteolin derivatives from oil palm (Elaeis guineensis Jacq.) leaf using LC-ESI-MS/MS. J Agric Food Chem. 2012;60(45):11201-10.

15. Tsivelika N, Irakli M, Mavromatis A, Chatzopoulou P, Karioti A. Phenolic Profile by HPLC-PDA-MS of Greek Chamomile Populations and Commercial Varieties and Their Antioxidant Activity. Foods. 2021;10(10).
16. Park SB, Song K, Kim YS. Tetra-cis/trans-Coumaroyl Polyamines as NK1 Receptor Antagonists from Matricaria chamomilla. Planta Med Int Open. 2017.
17. Yamamoto A, Nakamura K, Furukawa K, Konishi Y, Ogino T, Higashiura K, et al. A new nonpeptide tachykinin NK1 receptor antagonist isolated from the plants of Compositae. Chem Pharm Bull. 2002;50(1):47-52.
18. McNab H, Ferreira ESB, Hulme AN, Quye A. Negative ion ESI–MS analysis of natural yellow dye flavonoids—An isotopic labelling study. International Journal of Mass Spectrometry. 2008;284(1-3):57-65.
19. Slimen IB, Mabrouk M, Hanène C, Najar T, Abderrabba M. LC-MS Analysis of Phenolic Acids, Flavonoids and Betanin from Spineless Opuntia ficus-indica Fruits. Cell Biology. 2017;5(2).
20. Olennikov DN, Chirikova NK, Kashchenko NI, Nikolaev VM, Kim S-W, Venno C. Bioactive Phenolics of the Genus Artemisia (Asteraceae): HPLC-DAD-ESI-TQ-MS/MS Profile of the Siberian Species and Their Inhibitory Potential Against α-Amylase and α-Glucosidase. Frontiers in Pharmacology. 2018.
