# Supplementary material for: Comparative analysis of whole plant, flower and root extracts of Chamomilla recutita L. and characteristic pure compounds reveals differential anti-inflammatory effects on human T cells
Source: Front Immunol. 2024 Apr 24;15:1388962. doi: 10.3389/fimmu.2024.1388962 (PMC11077421; doi:10.3389/fimmu.2024.1388962)
Supplement: Supplementary file 3 [file Table_2.pdf]

Supplementary tables for nCounter gene expression and Ingenuity pathway analyses

Supplementary Table 2. List of significantly up and down regulated genes with corresponding log_2 fold ratios from comparison of different treatments against respective controls from nCounter analysis. CT: Chamomile aq. total ferm, CR: Chamomile aq. root ferm, CF: Chamomile ethanolic flower, CRE: Chamomile ethanolic root, CU: Chamomile mother tincture, Ap: Apigenin, Cz: Chamazulene

Sig genes (down – up)	CT vs none	CR vs none	CF vs EtOH	Ap vs DMSO	Cz vs DMSO
ABCB1			-0.176		
AHR					0.439
ARHGDIB					0.362
ATG16L1		0.293	0.384		
ATM			0.537		
BATF				-1.266	-0.573
BATF3				-1.335	
BAX				0.253	
BCL2	-0.213				
BCL2L11				-1.032	
BCL3			-0.586		
BST2			0.345		
CASP2				-0.381	
CASP3				-0.391	
CASP8			-0.164		
CCL20			-0.159	-1.094	-0.753
CCND3			-0.261		
CCR1			0.648		
CCRL1					-1.023
CD160			0.189		
CD22	-0.879				
CD27			0.267	1.198	0.350
CD274					-0.217
CD28			0.466		0.506
CD3D					
CD3E			0.368	0.466	0.340
CD3EAP				-0.669	
CD4		0.241			
CD40LG		-0.374	-0.226	-1.190	-0.405
CD44			0.205		0.322

Sig genes<br>down up	CT vs none	CR vs none	CF vs EtOH	Ap vs DMSO	Cz vs DMSO
CD45R0			-0.358		
CD45RA			0.242	0.828	0.300
CD45RB				0.686	
CD46					
CD48				0.344	
CD5					0.449
CD53					0.193
CD6				0.856	
CD7				0.371	0.214
CD81			0.144		
CD83				-0.791	
CD8B					-0.535
CD9		-0.500			
CD96	0.158			0.430	0.232
CD97			0.144	0.521	0.269
CDKN1A		0.420			
CEACAM1					-0.451
CEBPB	0.310				0.264
CFB					-0.290
CFD					-0.294
CHUK				-0.272	
CISH			0.366	-1.236	-0.586
CLEC6A		1.197			
CSF1				-1.470	
CSF2				-2.148	-0.755
CTLA4_all			0.149		
CTSC				-0.767	
CX3CL1		0.713			-0.697
CXCL10	-0.333			-0.529	
CXCL2		2.216			
CXCL9					-0.627
CXCR2		1.232			-0.600
CXCR3				-0.417	0.246
CXCR4					0.398
DEFB4A					-0.645
DUSP4		-0.207	-0.450	-0.568	
EDNRB		0.709			
EGR1			0.555		
EGR2				0.227	
ETS1					0.292

Sig genes (down — up)	CT vs none	CR vs none	CF vs EtOH	Ap vs DMSO	Cz vs DMSO
FCGR2B			0.507		
FKBP5				-0.692	
FYN			-0.132		
GBP1	-0.152				
GFI1			-0.531	-1.264	
GPI				-0.241	
GPR183			-0.202	-0.586	-0.390
GZMB				-1.702	-0.379
GZMK		0.610			
HLA-B				0.254	
HLA-C				0.371	
HLA-DMA					-0.310
HLA-DPA1				0.789	
HLA-DPB1					0.303
HRAS				-0.569	-0.372
ICAM1				-0.962	-0.538
ICAM2				0.413	
ICAM3				0.786	
IFI16				-0.448	
IFI35				-0.561	
IFITM1			-0.254		
IFNAR1		0.625			
IFNAR2					0.223
IFNG	-0.142			-1.360	
IGF2R			-0.458		
IKBKAP				-0.545	
IKBKG		0.283			
IKZF1					0.454
IL10				-1.440	-1.074
IL10RA					-0.280
IL13					-0.741
IL13RA1				0.688	
IL17A				-1.712	
IL17F				-1.292	
IL18R1					-0.339
IL1B		4.041			
IL1R1				-0.279	
IL1RAP			-0.559		
IL2	-0.340	-0.410	-0.324	-2.875	-1.215
IL21	-0.432			-1.402	-1.191

Sig genes (down → up)	CT vs none	CR vs none	CF vs EtOH	Ap vs DMSO	Cz vs DMSO
IL21R	-0.181			0.466	
IL22			-1.207	-2.558	-1.082
IL23A				-1.098	-0.459
IL2RA				0.151	-0.276
IL2RB		0.264			
IL2RG			0.092	0.224	0.138
IL3			-0.078		
IL4			-0.531		-0.817
IL5					-0.571
IL6ST			-0.183		-0.415
IL7R	0.196			0.513	0.554
IL8		3.291			
ILF3				0.423	
IRAK2				-0.405	
IRF4			0.359	0.280	
IRF8	-0.438			-1.245	-0.511
IRGM			0.815		
ITGA5				0.372	
ITGAL		0.444			
ITGB2					0.184
JAK2	-0.591				
JAK3			0.461		
KIR_Activating_Subgroup_1					-0.459
KIR_Inhibiting_Subgroup_2		0.789			
KIR3DL2			0.779		
KLRB1			0.292	0.777	
KLRC1		0.186			
KLRC2		0.777			
KLRD1				-0.733	
KLRG2					-0.876
LAG3				-0.645	
LCP2					-0.244
LIF				-1.058	-0.906
LILRB3			1.178		-0.556
LTA					-0.624
LY96					0.465
MAF					-0.459
MALT1	-0.189		-0.254	-0.823	
MAPK11		0.837			
MME			0.339		

Sig genes (down ↔ up)	CT vs none	CR vs none	CF vs EtOH	Ap vs DMSO	Cz vs DMSO
MX1	0.606				
MYD88			0.355	-0.802	
NCAM1	-0.447				
NCR1			1.059		
NFATC1				0.594	
NFATC3				-0.376	
NFKB1			-0.357		
NFKB2				0.249	
NFKBIA				0.286	
NOD2		0.999			
NOTCH1	-0.267			-0.701	
NOTCH2				-0.707	
PAX5		0.554			-0.757
PDCD2			0.307		
PDGFB		0.957			
PECAM1				-0.652	-0.301
PML		0.967			
PRKCD	0.333				
PSMB5		0.116	0.168		
PSMB7					
PSMB8			-0.440		
PSMD7				0.507	
PTGER4				0.166	
PTPN2			0.149		
PTPN22					0.530
PTPN6				-0.346	
RAF1			0.276		
RARRES3		0.374			
RELA			0.687		
RORC					
RUNX1		-0.219		-0.908	
S1PR1				-0.551	
SELPLG		0.448			
SH2D1A				-0.449	
SLAMF6			0.950		
SMAD3		0.347			
SOCS1				-0.665	
SOCS3				-1.124	-0.541
STAT4				-0.271	
STAT5B			0.329		0.285

Sig genes (down – up)	CT vs none	CR vs none	CF vs EtOH	Ap vs DMSO	Cz vs DMSO
TAGAP	-0.141		0.280	0.368	
TAP1	-0.136				
TCF7					0.347
TGFB1				-0.523	
TGFBI				0.859	
THY1	-0.750				-0.617
TICAM1				0.331	
TIGIT		0.296			
TLR9		1.580			
TNF	0.139				-0.332
TNFAIP3	-0.311		0.245		
TNFRSF13C				-0.401	
TNFRSF14					-0.346
TNFRSF4				-1.049	-0.336
TNFSF13B				0.802	
TNFSF8					-0.302
TP53				0.438	
TRAF1			0.374	0.212	
TRAF4			-0.160	-0.545	
TYK2			0.791		
UBE2L3		0.277			
VTN					-0.726
XCL1			0.290		
ZEB1		0.489			

Supplementary Table 3. Top Canonical pathways predicted to be up or down regulated by different extracts or pure compounds. CT: Chamomile aq. total ferm, CR: Chamomile aq. root ferm, CF: Chamomile ethanolic flower, CRE: Chamomile ethanolic root, CU: Chamomile mother tincture, Ap: Apigenin, Cz: Chamazulene

Extracts (Predicted upregulation or downregulation)

(A) CT 1:100 vs None

Ingenuity Canonical Pathways	*-log(p-value)*	*z-score*	*Molecules*
VDR/RXR Activation	8,1E00	-2,236	CEBPB,CXCL10,IFNG,IL2,PRKCD
Th1 Pathway	5,37E00	-2,000	IFNG,IL2,JAK2,NOTCH1
Th17 Activation Pathway	3,07E00	-2,000	IFNG,IL21,IL21R,JAK2
Macrophage Classical Activation Signaling Pathway	9,51E00	-1,890	CXCL10,IFNG,IL2,IL21,IRF8,JAK2,TNF
Pathogen Induced Cytokine Storm Signaling Pathway	7,56E00	-1,890	CXCL10,IFNG,IL2,IL21,IL21R,JAK2,TNF
Hepatic Fibrosis Signaling Pathway	4,45E00	0,447	BCL2,CEBPB,JAK2,PRKCD,TNF
Erythropoietin Signaling Pathway	7,94E00	0,816	IFNG,IL2,IL21,JAK2,PRKCD,TNF
PD-1, PD-L1 cancer immunotherapy pathway	5,6E00	1,000	IFNG,IL2,JAK2,TNF

(B) CR 1:100 vs None

Ingenuity Canonical Pathways	*-log(p-value)*	*z-score*	*Molecules*
IL-10 Signaling	4,12E00	-1,000	CDKN1A,IKBKG,IL1B,MAPK11
NOD1/2 Signaling Pathway	1,31E01	1,897	ATG16L1,CD40LG,CXCL8,IFNAR1,IKBKG,IL1B,IL2,MAPK11,NOD2,TLR9
TREM1 Signaling	5,36E00	2,000	CXCL8,IL1B,NOD2,TLR9
Role of MAPK Signaling in Inhibiting the Pathogenesis of Influenza	5,27E00	2,000	CXCL8,IL1B,MAPK11,PLAAT4
Role of Hypercytokinemia/hyperchemokinemia in the Pathogenesis of Influenza	5,17E00	2,000	CXCL8,IFNAR1,IL1B,TLR9
Immunogenic Cell Death Signaling Pathway	5,03E00	2,000	ATG16L1,GZMK,IFNAR1,IL1B
Osteoarthritis Pathway	4,65E00	2,000	CXCL8,CXCR2,IL1B,ITGAL,SMAD3
IL-33 Signaling Pathway	3,85E00	2,000	CXCL8,IKBKG,IL1B,MAPK11
Pulmonary Fibrosis Idiopathic Signaling Pathway	2,95E00	2,000	IL1B,MAPK11,PDGFB,SMAD3
Phagosome Formation	1,77E00	2,000	EDNRB,ITGAL,PLAAT4,TLR9
Pathogen Induced Cytokine Storm Signaling Pathway	1,16E01	2,111	CD40LG,CX3CL1,CXCL2,CXCL8,CXCR2,IFNAR1,IL1B,IL2,MAPK11,NOD2,TLR9
Cardiac Hypertrophy Signaling (Enhanced)	9,78E00	2,121	CD40LG,CXCL8,CXCR2,EDNRB,IFNAR1,IKBKG,IL1B,IL2,IL2RB,ITGAL,MAPK11
Role of Pattern Recognition Receptors in Recognition of Bacteria and Viruses	8,7E00	2,236	CD40LG,CLEC6A,CXCL8,IL1B,IL2,NOD2,TLR9
Neuroinflammation Signaling Pathway	5,19E00	2,236	CX3CL1,CXCL8,IKBKG,IL1B,MAPK11,TLR9
Senescence Pathway	4,19E00	2,236	CDKN1A,CXCL8,IKBKG,PML,SMAD3
G-Protein Coupled Receptor Signaling	2,51E00	2,236	CXCR2,DUSP4,EDNRB,IKBKG,MAPK11
Role of PKR in Interferon Induction and Antiviral Response	7,44E00	2,449	IFNAR1,IKBKG,IL1B,MAPK11,PDGFB,TLR9

S100 Family Signaling Pathway	3,16E00	2,449	CDKN1A,CXCL8,EDNRB,IL1B,MAPK11,SMAD3
FAK Signaling	2,51E00	2,449	CXCR2,EDNRB,IFNAR1,IL2RB,ITGAL,MAPK11
Neutrophil Extracellular Trap Signaling Pathway	6,93E00	2,828	CXCL8,CXCR2,IFNAR1,IL1B,ITGAL,MAPK11,PLAAT4, SELPLG

(C) CF 1:860 vs EtOH

Ingenuity Canonical Pathways	***-log(p-value)***	***z-score***	***Molecules***
Multiple Sclerosis Signaling Pathway	8,45E00	-3,000	CASP8,CD40LG,CTLA4,IL2,IL22,IL3,IL4,IRF4,NFKB1
Pathogen Induced Cytokine Storm Signaling Pathway	1,28E01	-2,138	CASP8,CCL20,CCR1,CD40LG,IL1RAP,IL2,IL22,IL3,IL4 ,IL6ST,MYD88,NFKB1,STAT5B,XCL1
IL-17 Signaling	6,52E00	-1,890	CCL20,CD40LG,IL2,IL3,IL4,NFKB1,RELA
Systemic Lupus Erythematosus In B Cell Signaling Pathway	1,22E01	-1,698	CCND3,CD40LG,FCGR2B,FYN,IL2,IL3,IL4,IL6ST,LILR B3,MALT1,MYD88,NFKB1,RAF1,RELA,TRAF1,TRAF4, TYK2
PD-1, PD-L1 cancer immunotherapy pathway	8,2E00	-1,633	CD28,IL2,IL2RG,IL4,JAK3,STAT5B,TYK2
Wound Healing Signaling Pathway	6,83E00	-1,414	CD40LG,IL1RAP,IL2,IL3,IL4,NFKB1,RAF1,TYK2
Th2 Pathway	1,54E01	0,577	CCR1,CD28,CD3E,GFI1,IL2,IL2RG,IL3,IL4,JAK3,NFKB 1,STAT5B,TYK2
IL-9 Signaling	1,18E01	1,633	BCL3,CISH,IL2RG,JAK3,NFKB1,RELA,STAT5B
Systemic Lupus Erythematosus In T Cell Signaling Pathway	3,1E00	1,633	CASP8,CD28,CD3E,CD40LG,CD44,IL2,IRF4
IL-10 Signaling	9,85E00	1,667	BCL3,CCR1,FCGR2B,IL1RAP,IL6ST,NFKB1,RELA,STA T5B,TYK2
ERB2-ERBB3 Signaling	4,78E00	2,000	JAK3,RAF1,STAT5B,TYK2
Erythropoietin Signaling Pathway	7,95E00	2,121	CD40LG,IL2,IL3,IL4,NFKB1,RAF1,RELA,STAT5B

Pure compounds (Predicted upregulation or downregulation)

(D) Ap 25 μM vs DMSO

Ingenuity Canonical Pathways	***-log(p-value)***	***z-score***	***Molecules***
IL-17 Signaling	1.13E+01	-2.887	CCL20,CD40LG,CSF2,HRAS,IFNG,IL17A,IL17F,IL2,IL21, LIF,TGFB1,TNFSF13B
Role Of Osteoblasts In Rheumatoid Arthritis Signaling Pathway	1.00E+01	-2.887	CD40LG,CSF2,CTSC,IFNG,IL17A,IL17F,IL2,IL21,LIF, STAT4,TGFB1,TNFSF13B
Wound Healing Signaling Pathway	1.36E+01	-2.84	CD40LG,CHUK,CSF2,HRAS,IFNG,IL17A,IL17F,IL1R1,IL 2, IL21,LIF,NFKB2,NFKBIA,TGFB1,TNFSF13B
Pathogen Induced Cytokine Storm Signaling Pathway	2.55E+01	-2.746	CASP3,CCL20,CD40LG,CSF2,CXCL10,CXCR3,GZMB, HLA-DPA1,IFNG,IL10,IL17A,IL17F,IL1R1,IL2,IL21, IL21R, IL22,IL23A,LIF,MYD88,NFKB2,SOCS3,STAT4,TGFB1, TICAM1,TNFSF13B
NOD1/2 Signaling Pathway	1.55E+01	-2.496	CD40LG,CHUK,CSF2,ELP1,IFNG,IL17A,IL17F,IL2,IL21,L IF,MYD88,NFKB2,NFKBIA,TGFB1,TNFSF13B
Macrophage Classical Activation Signaling Pathway	2.96E+01	-2.449	CCL20,CD40LG,CHUK,CSF2,CXCL10,HLA-DPA1,IFNG, IL10,IL13RA1,IL17A,IL17F,IL2,IL21,IL23A,IRF8,LIF,MYD8 8,NFKB2,NFKBIA,SOCS1,SOCS3,TGFB1,TICAM1, TNFSF13B
Th17 Activation Pathway	1.88E+01	-2.4	BATF,CCL20,CD3E,CSF2,IFNG,IL10,IL17A,IL17F,IL1R1, IL21,IL21R,IL22,IL23A,IRAK2,IRF4,MYD88,NFATC1, NFATC3,NFKB2,PTGER4,RUNX1,SOCS3,STAT4
Differential Regulation of Cytokine Production in Intestinal Epithelial Cells by IL-17A and IL-17F	7.74E+00	-2.236	CSF2,IFNG,IL10,IL17A,IL17F
IL-8 Signaling	4.87E+00	-2.236	BAX,CHUK,ELP1,HRAS,ICAM1,IRAK2,NFKBIA
HIF1α Signaling	2.95E+00	-2.236	GPI,HRAS,IL17A,TGFB1,TP53
IL-23 Signaling Pathway	1.70E+01	-2.111	CSF2,IL17A,IL17F,IL21,IL22,IL23A,NFKB2,NFKBIA, RUNX1,SOCS3,STAT4
HMGB1 Signaling	1.48E+01	-2.111	CD40LG,CSF2,HRAS,ICAM1,IFNG,IL17A,IL17F,IL1R1,IL 2, IL21,LIF,NFKB2,TGFB1,TNFSF13B

Differential Regulation of Cytokine Production in Macrophages and T Helper Cells by IL-17A and IL-17F	6.32E+00	-2	CSF2,IL10,IL17A,IL17F
VDR/RXR Activation	3.70E+00	-2	CSF2,CXCL10,IFNG,IL2
Erythropoietin Signaling Pathway	1.57E+01	1.807	CD40LG,CSF2,HRAS,IFNG,IL17A,IL17F,IL2,IL21,LIF,NFKB2,NFKBIA,PTPN6,TGFB1,TNFSF13B,TP53
CDX Gastrointestinal Cancer Signaling Pathway	1.09E+01	2.714	CD40LG,CHUK,CSF2,IFNG,IL17A,IL17F,IL2,IL21,LIF,NFKB2,TGFB1,TNFSF13B

(E) Cz 50 μM vs DMSO

Ingenuity Canonical Pathways	***-log(p-value)***	***z-score***	***Molecules***
Pathogen Induced Cytokine Storm Signaling Pathway	3,17E01	-3,402	CCL20,CD40LG,CSF2,CX3CL1,CXCL9,CXCR2,CXCR3,CXCR4,GZMB,HLA-DMA,HLA-DPB1,IFNAR2,IL10,IL13,IL18R1,IL2,IL21,IL22,IL23A,IL4,IL5,IL6ST,LIF,LTA,SOCS3,STAT5B,TNF,TNFSF8
IL-17 Signaling	1,71E01	-3,357	CCL20,CD40LG,CEBPB,CSF2,DEFB4A/DEFB4B,HRAS,IL13,IL2,IL21,IL4,IL5,LIF,LTA,TNF,TNFSF8
Role Of Osteoblasts In Rheumatoid Arthritis Signaling Pathway	1,4E01	-3,207	CD40LG,CSF2,IL13,IL2,IL21,IL4,IL5,IL6ST,LIF,LTA,STAT5B,TCF7,TNF,TNFSF8
HMGB1 Signaling	1,47E01	-3,162	CD40LG,CSF2,HRAS,ICAM1,IL13,IL2,IL21,IL4,IL5,LIF,LTA,TNF,TNFSF8
Systemic Lupus Erythematosus In B Cell Signaling Pathway	1,06E01	-3,153	CD40LG,CD5,CSF2,HRAS,IFNAR2,IL10,IL13,IL2,IL21,IL4,IL5,IL6ST,LIF,LILRB3,LTA,TNF,TNFSF8
Wound Healing Signaling Pathway	1,23E01	-3,051	CD40LG,CEBPB,CSF2,HRAS,IL13,IL2,IL21,IL4,IL5,LIF,LTA,TNF,TNFSF8
Cardiac Hypertrophy Signaling (Enhanced)	1,61E01	-3,000	CD40LG,CSF2,CXCR2,HRAS,IL10RA,IL13,IL18R1,IL2,IL21,IL2RA,IL2RG,IL4,IL5,IL6ST,IL7R,ITGB2,LIF,LTA,TNF,TNFSF8
NOD1/2 Signaling Pathway	1,26E01	-2,887	CD40LG,CSF2,IFNAR2,IL13,IL2,IL21,IL4,IL5,LIF,LTA,TNF,TNFSF8
Macrophage Classical Activation Signaling Pathway	2,86E01	-2,837	CCL20,CD40LG,CSF2,CXCL9,HLA-DMA,HLA-DPB1,IFNAR2,IL10,IL13,IL2,IL21,IL23A,IL4,IL5,IRF8,LIF,LTA,LY96,MAF,SOCS3,TNF,TNFSF8
Multiple Sclerosis Signaling Pathway	2,04E01	-2,828	CD40LG,CSF2,HLA-DMA,HLA-DPB1,IL10,IL13,IL2,IL21,IL22,IL23A,IL2RA,IL4,IL5,IL7R,LIF,LTA,TNF,TNFSF8
Dendritic Cell Maturation	5,94E00	-2,530	CD3E,CD40LG,CSF2,HLA-DMA,HLA-DPB1,ICAM1,IL10,IL23A,IRF8,LTA,TNF
IL-33 Signaling Pathway	4,73E00	-2,449	CSF2,ICAM1,IL13,IL4,IL5,TNF
Neuroinflammation Signaling Pathway	4,3E00	-2,449	CX3CL1,HLA-DMA,HLA-DPB1,ICAM1,IL10,IL4,TNF
Differential Regulation of Cytokine Production in Intestinal Epithelial Cells by IL-17A and IL-17F	8,24E00	-2,236	CSF2,DEFB4A/DEFB4B,IL10,IL13,TNF
Differential Regulation of Cytokine Production in Macrophages and T Helper Cells by IL-17A and IL-17F	6,71E00	-2,000	CSF2,IL10,IL13,TNF
Inhibition of ARE-Mediated mRNA Degradation Pathway	2,9E00	-2,000	CD40LG,LTA,TNF,TNFSF8
Natural Killer Cell Signaling	2,54E00	-2,000	HRAS,IL18R1,IL2,LCP2
NF-κB Signaling	1,6E00	-2,000	CD3E,CD40LG,HRAS,LTA,TNF
Tumor Microenvironment Pathway	9,76E00	-1,897	CD274,CD44,CSF2,CXCR4,HRAS,ICAM1,IL10,IL13,IL4,TNF
Th17 Activation Pathway	6,82E00	-1,897	AHR,BATF,CCL20,CD3E,CSF2,DEFB4A/DEFB4B,IL10,IL21,IL22,IL23A,SOCS3
T Cell Exhaustion Signaling Pathway	8,04E00	-1,667	BATF,CD274,CD28,CD3E,GZMB,HLA-DMA,HLA-DPB1,HRAS,IFNAR2,IL10,IL10RA,TCF7,TNFRSF14
IL-23 Signaling Pathway	8,39E00	-1,633	CSF2,IL21,IL22,IL23A,SOCS3,TNF
CDX Gastrointestinal Cancer Signaling Pathway	1,21E01	2,887	CD40LG,CSF2,IL13,IL2,IL21,IL4,IL5,LIF,LTA,TCF7,TNF,TNFSF8
Erythropoietin Signaling Pathway	1,42E01	3,051	CD40LG,CSF2,HRAS,IL13,IL2,IL21,IL4,IL5,LIF,LTA,STAT5B,TNF,TNFSF8
